# IL-7 Receptor Mutations and Steroid Resistance in Pediatric T cell Acute Lymphoblastic Leukemia: A Genome Sequencing Study

**DOI:** 10.1371/journal.pmed.1002200

**Published:** 2016-12-20

**Authors:** Yunlei Li, Jessica G. C. A. M. Buijs-Gladdines, Kirsten Canté-Barrett, Andrew P. Stubbs, Eric M. Vroegindeweij, Willem K. Smits, Ronald van Marion, Winand N. M. Dinjens, Martin Horstmann, Roland P. Kuiper, Rogier C. Buijsman, Guido J. R. Zaman, Peter J. van der Spek, Rob Pieters, Jules P. P. Meijerink

**Affiliations:** 1 Department of Pediatric Oncology/Hematology, Erasmus Medical Center/Sophia Children’s Hospital, Rotterdam, The Netherlands; 2 Princess Máxima Center for Pediatric Oncology, Utrecht, The Netherlands; 3 Department of Bioinformatics, Erasmus Medical Center, Rotterdam, The Netherlands; 4 Department of Pathology, Erasmus Medical Center, Rotterdam, The Netherlands; 5 Research Institute Children’s Cancer Center Hamburg, Hamburg, Germany; 6 Clinic of Pediatric Hematology and Oncology, University Medical Center Hamburg-Eppendorf, Hamburg, Germany; 7 Co-operative Study Group for Childhood Acute Lymphoblastic Leukemia, Hamburg, Germany; 8 Department of Human Genetics, Radboud University Medical Center, Nijmegen, The Netherlands; 9 Netherlands Translational Research Center, Oss, The Netherlands; MSKCC, UNITED STATES

## Abstract

**Background:**

Pediatric acute lymphoblastic leukemia (ALL) is the most common childhood cancer and the leading cause of cancer-related mortality in children. T cell ALL (T-ALL) represents about 15% of pediatric ALL cases and is considered a high-risk disease. T-ALL is often associated with resistance to treatment, including steroids, which are currently the cornerstone for treating ALL; moreover, initial steroid response strongly predicts survival and cure. However, the cellular mechanisms underlying steroid resistance in T-ALL patients are poorly understood. In this study, we combined various genomic datasets in order to identify candidate genetic mechanisms underlying steroid resistance in children undergoing T-ALL treatment.

**Methods and Findings:**

We performed whole genome sequencing on paired pre-treatment (diagnostic) and post-treatment (remission) samples from 13 patients, and targeted exome sequencing of pre-treatment samples from 69 additional T-ALL patients. We then integrated mutation data with copy number data for 151 mutated genes, and this integrated dataset was tested for associations of mutations with clinical outcomes and in vitro drug response. Our analysis revealed that mutations in *JAK1* and *KRAS*, two genes encoding components of the interleukin 7 receptor (IL7R) signaling pathway, were associated with steroid resistance and poor outcome. We then sequenced *JAK1*, *KRAS*, and other genes in this pathway, including *IL7R*, *JAK3*, *NF1*, *NRAS*, and *AKT*, in these 69 T-ALL patients and a further 77 T-ALL patients. We identified mutations in 32% (47/146) of patients, the majority of whom had a specific T-ALL subtype (early thymic progenitor ALL or TLX). Based on the outcomes of these patients and their prednisolone responsiveness measured in vitro, we then confirmed that these mutations were associated with both steroid resistance and poor outcome.

To explore how these mutations in IL7R signaling pathway genes cause steroid resistance and subsequent poor outcome, we expressed wild-type and mutant IL7R signaling molecules in two steroid-sensitive T-ALL cell lines (SUPT1 and P12 Ichikawa cells) using inducible lentiviral expression constructs. We found that expressing mutant IL7R, JAK1, or NRAS, or wild-type NRAS or AKT, specifically induced steroid resistance without affecting sensitivity to vincristine or *L*-asparaginase. In contrast, wild-type IL7R, JAK1, and JAK3, as well as mutant JAK3 and mutant AKT, had no effect. We then performed a functional study to examine the mechanisms underlying steroid resistance and found that, rather than changing the steroid receptor’s ability to activate downstream targets, steroid resistance was associated with strong activation of MEK-ERK and AKT, downstream components of the IL7R signaling pathway, thereby inducing a robust antiapoptotic response by upregulating MCL1 and BCLXL expression. Both the MEK-ERK and AKT pathways also inactivate BIM, an essential molecule for steroid-induced cell death, and inhibit GSK3B, an important regulator of proapoptotic BIM. Importantly, treating our cell lines with IL7R signaling inhibitors restored steroid sensitivity. To address clinical relevance, we treated primary T-ALL cells obtained from 11 patients with steroids either alone or in combination with IL7R signaling inhibitors; we found that including a MEK, AKT, mTOR, or dual PI3K/mTOR inhibitor strongly increased steroid-induced cell death. Therefore, combining these inhibitors with steroid treatment may enhance steroid sensitivity in patients with ALL. The main limitation of our study was the modest cohort size, owing to the very low incidence of T-ALL.

**Conclusions:**

Using an unbiased sequencing approach, we found that specific mutations in IL7R signaling molecules underlie steroid resistance in T-ALL. Future prospective clinical studies should test the ability of inhibitors of MEK, AKT, mTOR, or PI3K/mTOR to restore or enhance steroid sensitivity and improve clinical outcome.

## Abstract 2

Jules Meijerink and colleagues study mechanisms of steroid resistance in pediatric T-cell acute lymphoblastic leukemia.

## Author Summary

**Why Was This Study Done?:**

- Although modern treatment protocols have drastically increased the cure rate among patients with T cell acute lymphoblastic leukemia (T-ALL), nearly 40% of patients require the most aggressive treatment regimen, significantly increasing the risk of harmful treatment effects later in life. These detrimental effects can include growth defects, bone necrosis, heart failure, and an increased risk of developing secondary malignancies. Moreover, treatment outcome for relapsed T-ALL patients is extremely poor.
- Steroids are the cornerstone chemotherapeutic drug in the treatment of acute lymphoblastic leukemia (ALL), including T-ALL. However, steroid resistance is common among patients and is associated with poor outcome and an increased risk of relapse.
- The mechanisms underlying steroid resistance in patients with ALL are poorly understood.
- Therefore, we performed an unbiased, comprehensive genetic analysis of pediatric T-ALL, as well as in vitro functional analyses to validate associations between the identified mutations and steroid resistance.

**What Did the Researchers Do and Find?:**

- We performed whole genome and targeted exome sequencing in patients with T-ALL and identified mutations in 151 genes, many of which are involved in cytokine signaling, transcriptional regulation, cell death, cell cycle, chromatin modification, and cellular transport.
- Mutation data were integrated with changes in chromosomal copy number and were correlated with the patients’ clinical features and underlying biological characteristics. Mutations in the IL7R signaling components *JAK1* and *KRAS* were correlated with steroid resistance and poor outcome.
- Sequencing of IL7R signaling molecules in a larger pediatric T-ALL cohort revealed mutations in 32% of patients.
- Expressing specific mutant and/or wild-type IL7R signaling molecules in two steroid-sensitive T-ALL cell lines induced steroid resistance via robust downstream signaling through MEK-ERK and AKT, thereby reducing steroid-induced apoptosis. Moreover, treating these cells with inhibitors of IL7R signaling restored steroid sensitivity.
- Primary T-ALL cells obtained from patients were treated with steroids either alone or in combination with IL7R signaling inhibitors. We found that including these inhibitors significantly enhanced steroid-induced cell death.

**What Do These Findings Mean?:**

- These results should be tested further in prospective patient cohorts, to investigate the possibility that including IL7R signaling inhibitors in treatment regimens could restore or enhance steroid sensitivity in patients with ALL, thereby improving clinical outcomes.

## Introduction

In children with acute lymphoblastic leukemia (ALL), response to therapy, including in vitro or in vivo steroid response, is a strong predictor of survival and cure [1–3]. ALL can be classified as T cell ALL (T-ALL) or B cell precursor ALL (BCP-ALL): T-ALL, particularly, has a high risk of relapse and is refractory to further treatment due to acquired therapy resistance. The mechanisms that underlie steroid resistance are poorly understood. In contrast to cell lines, which often harbor mutations and/or deletions in the steroid receptor NR3C1 [4], mutations are relatively rare among patients with ALL [5,6]. Upon steroid binding, NR3C1 translocates to the nucleus and drives the expression of target genes [7]. To date, steroid resistance has not been associated with reduced *NR3C1* expression, expression of *NR3C1* splice variants [8–10], or reduced expression of chaperone proteins [11,12]. Therefore, steroid resistance seems to be independent of changes in the *NR3C1* gene itself in most patients with steroid-resistant T-ALL. Several mechanisms have been proposed to explain steroid resistance in T-ALL including activation of AKT1, which phosphorylates serine 134 of NR3C1, thereby preventing nuclear translocation [13]. Also, elevated MYB and BCL2 concentrations may promote survival following steroid treatment [14]. Activated NOTCH1 may confer steroid resistance by repressing expression of *NR3C1* and *PTEN* [15]. Mutations in *RAS* have been shown to be associated with steroid resistance in BCP-ALL and are prevalent in relapsed patients [16–18]. Recently, CASP1 and its activator, NLRP3, were also shown to be associated with steroid resistance in ALL [19].

In this study, we aimed to provide an unbiased and comprehensive analysis of the molecular mechanisms that drive T-ALL and to resolve the cellular mechanisms that underlie steroid resistance. For this, we performed whole genome sequencing (WGS) and targeted exome sequencing (TES) in diagnostic patient samples obtained from pediatric T-ALL patients. Mutation data were integrated with copy number changes as determined by array comparative genomic hybridization (aCGH) to capture the full complexity of genomic mutations in T-ALL. Identification of steroid resistance mechanisms may provide therapeutic treatment options to improve sensitivity to this cornerstone chemotherapeutic drug in ALL treatment, improve cure rates, and help reduce detrimental late side effects of intensive treatment schedules through dose reduction.

## Methods

### Study Outline

**Fig 1:**
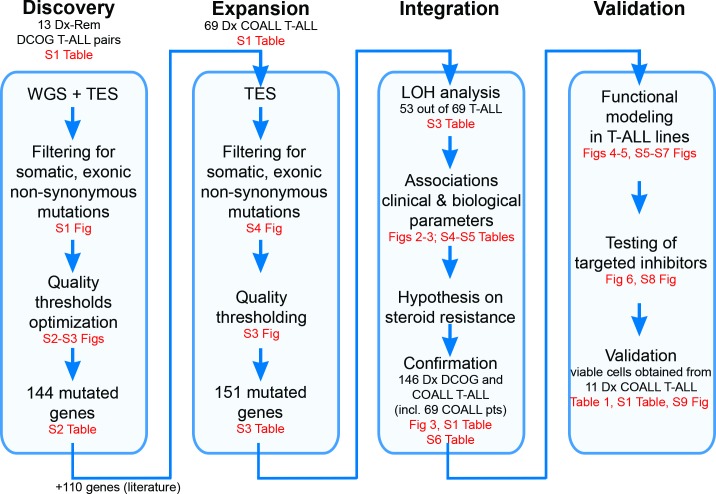
Study overview. Discovery phase: Whole genome sequencing (WGS) followed by targeted exome sequencing (TES) and prioritization of high-confidence mutations in 13 paired diagnostic (Dx)–remission (Rem) T cell acute lymphoblastic leukemia (T-ALL) patient samples. Expansion phase: TES for 254 genes on diagnostic materials of 69 T-ALL patients. Integration phase: Integration of high-confidence TES mutation and array comparative genomic hybridization loss of heterozygosity (LOH) datasets and associations with clinical and biological data. Confirmation of findings was done using an extended cohort of 146 diagnostic T-ALL patient samples, including the 69 patients (pts) in the expansion phase and 77 additional patients. Validation phase: Functional modeling in T-ALL cell lines, determination of efficacy of targeted inhibitors to revert phenotype, and testing in primary T-ALL patient samples. COALL, Co-operative Study Group for Childhood Acute Lymphoblastic Leukemia; DCOG, Dutch Childhood Oncology Group.

This study did not have a protocol or prospective analysis plan. An outline of this study is provided in Fig 1. Briefly, to obtain insight into the genetic landscape of pediatric T-ALL, we performed WGS on paired diagnostic–remission samples from 13 patients covering all of the most predominant genetic subtypes in T-ALL. Recurrence of identified mutations was then established by applying a TES approach to a cohort of diagnostic samples from 69 well-characterized pediatric T-ALL patients, and these mutation data were further integrated with copy number data for mutant genes as obtained by aCGH. The mutation/aberration statuses of 151 genes that were identified were then correlated with the patients’ clinical features and underlying biological characteristics including in vitro drug response, T-ALL subtype, and outcome. We found that mutations in components (*KRAS* and *JAK1*) of the IL7R signaling pathway correlated with steroid resistance and poor outcome. We then used a PCR–Sanger sequencing approach to identify mutations in other IL7R signaling components, including *IL7R*, *JAK1*, *JAK3*, *NF1*, *NRAS*, *KRAS*, and *AKT* genes, in an expanded cohort of diagnostic patient samples including these 69 patients and 77 additional T-ALL patients. The association between mutant signaling components and steroid resistance was then functionally explored in two steroid-sensitive T-ALL cell lines (SUPT1 and P12 Ichikawa), and the ability of IL7R signaling inhibitors to revert steroid resistance in these models was tested. Moreover, the ability of these inhibitors to restore or enhance steroid sensitivity was then investigated in primary leukemic cells isolated from 11 T-ALL patients.

### Patient Samples

Diagnostic primary leukemia samples from 162 pediatric T-ALL patients were used for this study (Fig 1; S1 Table). In the discovery phase, we used DNA isolated from matched pre-treatment (diagnostic) and post-treatment (remission) sample pairs from 13 pediatric T-ALL patients who enrolled in the Dutch Childhood Oncology Group (DCOG) ALL-10 protocol between 2004 and 2012. In the expansion phase of this study, we used DNA from diagnostic, pre-treatment patient material from 69 patients who enrolled in the German Co-operative Study Group for Childhood Acute Lymphoblastic Leukemia (COALL) protocol between 1997 and 2003 (COALL-97). Initial findings were then confirmed in a larger cohort of diagnostic, pre-treatment T-ALL patient materials including DNA from the previously mentioned 69 COALL patients plus that of five additional patients who also enrolled in the COALL-97 protocol and 72 additional T-ALL patients who enrolled in the DCOG protocols ALL-7/8 (*n* = 30) or ALL-9 (*n* = 42). Functional validation was done on viably frozen pre-treatment leukemia cells from peripheral blood or bone marrow from eight of the 74 patients who enrolled in the COALL-97 protocol (as mentioned above) plus two additional patients who enrolled in the COALL-03 study and one patient who enrolled in the DCOG ALL-10 study. Median follow-up for patients who enrolled in the DCOG ALL-7/8/9 (1988–2004) and COALL-97 protocols was 67 and 52 mo, respectively. The patients’ parents or legal guardians provided informed consent to use leftover diagnostic material for research with approval from the institutional review board of the Erasmus MC Rotterdam and in accordance with the Declaration of Helsinki. Leukemia cells were harvested from blood or bone marrow samples and enriched to a purity of at least 90% as described previously [20].

### Whole Genome Sequencing

Sequencing of 13 T-ALL tumor pairs in the discovery cohort (S1 and S2 Tables) was performed at Complete Genomics (Mountain View, California) using unchained combinatorial probe-anchor ligation chemistry on arrays of self-assembling DNA nanoballs producing 35-bp paired-end reads [21]. The average gross mapping yield for the 26 genomes was 183 Gb. On average 96.5% of the genome was called with 55× coverage or higher. Structural variants in the whole-genome-sequenced DNA of the 13 patients were detected using Complete Genomics’ cgatools (version 2.0.2.17) and were compared to the NCBI reference genome build 37. For this, junctions were identified that were not adjacent according to the reference sequence. Subsequently, somatic junctions were discovered as junctions detected in the tumor samples that were absent in the matching normal samples. Finally, we filtered for somatic high-confidence junctions using the following criteria: (1) there are at least ten mate pairs in the discordant reads cluster; (2) the de novo assembly of the junction is successful; (3) the junction exhibits high mapping diversity, i.e., the distance between the first position of the left-most mate read and the last position of the right-most mate read in the discordant reads cluster is more than 70 bp; (4) known underrepresented repeat sequences are not involved, e.g., ALR/Alpha; (5) the variant is absent in dbSNP build 132; (6) the variant does not result from deletion events of transposable elements (AluY and L1 subclasses); and (7) the variant is absent in 52 normal genomes that served as the baseline reference set (ftp://ftp2.completegenomics.com/Baseline_Genome_Set/SVBaseline/).

For mutation detection, reads were aligned to the NCBI build 36 reference genome by applying a local de novo assembly approach, and variations were called using Complete Genomics software v1.8 and v1.12. In each sample, 3.4 × 10^6^ single nucleotide variants (SNVs), 221.7 × 10^3^ small insertions, 234.5 × 10^3^ small deletions, and 77.3 × 10^3^ substitutions were detected on average. For this study, we focused on tumor-specific, non-synonymous mutations in exons, excluding sequence polymorphisms as present in NCBI dbSNP databases or the 1000 Genomes Project databases. This revealed 460 SNVs and 808 small insertions or deletions (INDELs) (S1 Fig) in the exons of the 13 T-ALL patients in total. To optimize thresholds for Complete Genomics quality parameters, we validated 46 mutations by PCR and Sanger sequencing. We found two quality parameters informative to distinguish true from falsely called mutations (S2A Fig). One is total score (TS), representing the confidence in the called mutation. The other is somatic score (SS), representing the confidence that the mutation is present in the tumor and absent in the matched remission sample. These two scores were calculated using Complete Genomics software v1.8 and v1.12 and CGA Tools 1.4.0. Based on the receiver operating characteristic (ROC) curves (S2B Fig), SS ≥ 0.1 and TS ≥ 100 were used as thresholds to reliably call somatic mutations with high confidence. Based on these thresholds, 137 genes were found to carry 178 high-confidence SNV or INDEL mutations (S1 Fig). The WGS sequence data of 26 genomes, aligned to the human reference genome (NCBI build 36), have been deposited in the European Nucleotide Archive with accession numbers ERS934791–ERS934816.

### PCR Amplification and Sanger Sequencing

PCR reactions were performed using 25–50 ng of genomic DNA, 300 nM primers, 200 μM dNTPs, 2 mM MgCl_2_, and 1.25 units of AmpliTaq Gold (Applied Biosystems) in 1× PCR Buffer II (Applied Biosystems) in a volume of 50 μl. PCR products were purified with the Millipore Vacuum Manifold filter system and sequenced (BigDye Terminator v3.1 Cycle Sequencing Kit, Applied Biosystems) on the ABI PRISM 3130 DNA Analyzer (Applied Biosystems).

### Targeted Exome Sequencing

To compare WGS with TES variant calling and to validate and determine quality parameters for WGS and TES, respectively, we performed TES for 410 mutated genes as found by WGS in the diagnostic samples of the 13 discovery cohort patients (S1 and S2 Tables). These genes included all 137 genes with high-confidence mutations and 273 mutated genes that had lower WGS quality scores.

Enrichment of exonic DNA sequences from genomic DNA was performed using Agilent SureSelect MP4 arrays (2-Mb capture region, with >2× average coverage). Sequencing was performed at ServiceXS. (Leiden, The Netherlands) using the Illumina HiSeq 2000 platform, which produced 100-bp paired-end reads. Image analysis, base calling, and quality check were performed using the Illumina data analysis pipeline RTA v1.13.48 and/or OLB v1.9 and CASAVA v1.8.2. Prior to alignment, reads were filtered based on the following quality thresholds: read trimming at Phred quality score 20 and minimal read length 36. The reads were then aligned to the NCBI build 37 reference genome using a short read aligner based on Burrows-Wheeler Transform [22] with a mismatch rate of 4%. SNV and INDEL mutations were identified using a ServiceXS in-house pipeline based on Bayesian statistics with the following thresholds: minimal coverage of 5× for INDELs and 10× for SNVs, with a minimal variant frequency of 30%.

Mutations detected by both WGS and TES platforms can be regarded as true mutations and hence helped to define the quality threshold parameters for the TES approach. More than 60% of predicted exonic, non-synonymous SNVs identified by TES had not been identified by WGS (S3 Fig). Exploiting the overlapping mutations detected by both platforms, we trained a one-class classifier to determine the quality parameter boundaries for mutation detection by TES. The TES quality parameters include read depth and the variant overall quality score. For SNV calling, we trained a Gaussian one-class classifier using ddtools [23] (S3A Fig). The performance of the Gaussian classifier is presented by the ROC curve in S3B Fig. The boundary was chosen to call mutations with a false negative rate (FNr) of 0.10, resulting in a false positive rate (FPr) of 0.28. Given the distribution of the INDEL training set as shown in S3C Fig, a simple decision tree classifier for calling INDEL mutations was built that resulted in a FNr of 0.10 and a FPr of 0.63, confirming the low concordance of INDEL mutations by both platforms.

To evaluate the recurrence of 137 high-confidence mutated genes and seven validated low-confidence mutated genes as identified by WGS (S2 Table), we sequenced the diagnostic samples of 69 genetically and clinically well-annotated T-ALL patients by TES for these 144 genes, together with an additional set of 110 genes that are recurrently mutated in leukemia [24,25] (S1 and S3 Tables). The mean coverage by TES was 561×, and 89% of all captured exons were covered by more than 100 reads. A stringent filtering strategy, as used for WGS (S4 Fig), was then applied to the TES results. Polymorphisms present in an expanded panel of genetic variation databases (listed in the next section) were excluded, providing an effective filter to exclude germline variants in the expanded cohort of 69 T-ALL patients for whom paired normal samples were not available [26]. The TES sequence data of the 13 discovery cohort patients and the 69 expansion cohort patients have been aligned to the human reference genome (NCBI build 37) and are deposited in the European Nucleotide Archive with accession numbers ERS935731–ERS935812.

### Genetic Variation Databases for TES

We increased the number of genetic variation databases to filter out polymorphisms from the mutation dataset as determined by TES by including dbSNP [27] versions 130, 131, 132, and 135; 1000 Genomes Project database [28] versions 2010 November and 2012 April; the ESP6500 database (http://evs.gs.washington.edu/EVS/); the Core and Diversity Panels in Huvariome [29], and an in-house dataset of 1,302 exomes from Radboud University Medical Center, Nijmegen, The Netherlands [30]. In addition, we excluded mutations that were also identified in the 13 remission samples by WGS. We retained mutations that represented polymorphisms according to the databases mentioned above when variations involving the same amino acids were annotated in the COSMIC database. By applying stringent filtering against a broad range of genetic variation databases, we aimed to remove as many germline variants as possible. To segregate germline polymorphisms from somatic mutations in patient samples in the absence of paired normal control samples, variations observed in thousands of unrelated individuals provide an effective filter [26].

### Copy Number Aberrations

We performed copy number analysis based on aCGH using SurePrint G3 Human CGH 2×400K arrays (Agilent Technologies) in diagnostic leukemia samples of 53 out of the 69 expansion cohort patients whose genomes were sequenced by TES (S3 Table). The array images were processed to obtain the log10 ratio of red and green channel signals, after background correction and dye normalization using Agilent Feature Extraction software (version 10.5.1.1). The probe chromosomal locations were annotated using the NCBI reference genome build 36. Afterwards, the log ratios were subjected to a noise-reduction algorithm, the Waves aCGH Correction Algorithm [31], to reduce the wave artifact characterized by an undulating aCGH profile along the chromosome. This algorithm corrects biases that are caused by differences in labeling intensities of DNA fragments, which depend on GC content and fragment size as well as the hybridization efficiencies of labeled fragments to corresponding hybridization probes on the array. Afterwards, the log10 ratios (*L*) were converted to copy number (CN) as CN = 2 × 10^*L*^. Amplifications and deletions were then called if three consecutive probe sets in a gene had a CN value beyond average CN ± 2 × the standard deviation of CN of the corresponding probe sets among all samples.

### SNP Array Analysis

The allele-specific copy number of the diagnostic sample from patient #10793 was obtained using Affymetrix SNP Array 6.0 with 680-bp median intermarker spacing. For this, 500 ng of genomic DNA isolated from the diagnostic and remission samples was used as input. Raw signal intensities were analyzed using Partek Genomics Suite. First, probe intensities were adjusted for a number of properties that are correlated with intensity, including fragment length, GC content, and other sequence-based hybridization bias. After quantile normalization, paired analysis was performed to generate allele-specific copy numbers by comparing the diagnostic sample to the remission sample.

### Pathway Enrichment Analysis

Pathway enrichment analysis was performed on 127 genes that had mutations or copy number aberrations in more than one patient sample in the expansion cohort. Two approaches were applied. One used the Database for Annotation, Visualization and Integrated Discovery (DAVID) v6.7. The functional annotation clustering reports of enriched biological pathways and molecular functions annotated by Gene Ontology can be found in S4 Table. The other approach used Ingenuity Pathway Analysis to identify enriched canonical pathways in these 127 recurrent genes. Default settings were applied except for setting the species to human only. The *p*-values indicated are calculated using a two-sided Fisher’s exact test.

### Gene Expression Profiling

The gene expression profiling dataset of the 117 pediatric T-ALL cases as produced by microarray [32] is available at GEO (http://www.ncbi.nlm.nih.gov/geo/) under accession number GSE26713.

### Statistical Tests

For the 69 COALL cohort samples in the expansion phase, we related the mutational statuses and copy number aberrations in 151 genes (S3 Table) to three clinical/biological parameters: T-ALL subtype (as defined previously [32]), in vitro prednisolone LC50 level, and survival outcome. In these analyses, the presence of mutations (Mut), deletions (Del), amplifications (Amp), and the combinations MutDel, MutAmp, or MutAber (i.e., mutations and/or copy number aberrations including deletions and amplifications) in each gene was tested against the clinical/biological parameters. Each gene with a specific feature (e.g., JAK1_Mut) was tested individually. The features are binary (present/absent), which means multiple incidences of the same feature in the same gene for the same patient sample are aggregated. For example, JAK1_Mut = “present” in a patient sample means that there is at least one JAK1 mutation identified in the sample. Associations between any of these six features and T-ALL subtype were calculated using Fisher’s exact test. Associations with in vitro prednisolone LC50 were calculated using the Kruskal-Wallis test. All *p*-values are two-sided. Associations with survival were calculated using the log-rank test. For all test results, *p*-values were used to prioritize our hypotheses on associations and therefore remained nominal without multiple testing corrections. A threshold of nominal *p <* 0.05 was used to present findings that are potentially more relevant. Using this threshold, genes that are associated with reduced drug sensitivity, poor event-free or relapse-free survival, or particular T-ALL subtypes are summarized in S5 Table. Given the data availability of the patient samples (S1 Table), the number of tests varies for different associations and is indicated in S5 Table.

In the integration phase, we validated the associations identified in the expansion phase. For this, we performed PCR–Sanger sequencing for *IL7R*, *JAK1*, *JAK3*, *NF1*, *NRAS*, *KRAS*, and *AKT* genes (i.e., IL7R signaling pathway genes) in the confirmation cohort of 146 patients comprising the 69 patients from the expansion phase and 77 additional pediatric T-ALL patients. The presence of mutations in these genes as well as available information on genetic aberrations affecting the *PTEN* gene were used to segregate patients into three groups, i.e., those with mutations in the IL7R signaling pathway, those with mutations in PTEN, and the rest of the patients. Association between IL7R pathway mutations and T-ALL subtype was tested by Fisher’s exact test. Association between IL7R pathway mutations and in vitro prednisolone LC50 was tested by Kruskal-Wallis test in 97 patients for whom in vitro prednisolone response data were available. Association between IL7R pathway mutations and survival was tested using the log-rank test.

### Gateway Cloning of Lentiviral Expression Vectors and Virus Production

Gateway multi-site recombination (Invitrogen) was used to simultaneously clone multiple DNA fragments into our Gateway-adapted lentiviral pLEGO-iC2 destination vector (Addgene). First, the parental pLEGO-iC2 vector was converted into a Gateway destination vector by replacing the pSFFV-MCS-IRES-mCherry insert with the Gateway Cassette A. Cassette A, comprising the *ccdB* and chloramphenicol resistance genes flanked by *attR* recombination sites, was amplified by PCR and cloned into the ApaI/PciI-digested pLEGO-iC2 vector. Lentiviral expression vectors were assembled using Gateway recombination of the lentiviral destination vector with four synthetically synthesized entry vectors (Eurogentec): (1) *attL1/attR5*-flanked doxycycline-inducible promoter (third generation, Clontech); (2) *attL5/attL4*-flanked human cDNA sequence; (3) *attR4/attR3*-flanked DDK-tag followed by a stop codon, WPRE sequences, and a constitutive pSFFV promoter; and (4) *attL3/attL2*-flanked TETon-T2A (*Thosea asigna* virus 2A peptide)–puromycin resistance cassette or TETon-T2A-LNGFR (truncated or ΔNGFR) reporter. LR-recombination reactions were performed according to the manufacturer’s instructions. For the shRNA experiments, PLKO.1-puro lentiviral shRNA constructs directed against the human *NR3C1* gene were selected from the MISSION T shRNA Library (Sigma-Aldrich). For lentivirus production, HEK293T cells were transfected with lentiviral expression vector DNA and pMD2.G (VSV-G), pMDLg/pRRE, and pRSV-REV support vectors (Addgene) using X-tremeGENE HP DNA Transfection Reagent (Roche). Transfection was performed in DMEM supplemented with 10% heat-inactivated fetal calf serum (FCS), 1× Glutamax, 1% penicillin/streptomycin, and 0.25 μg/ml Fungizone, and the HEK293T cells were cultured overnight in a humidified incubator at 37°C and 5% CO_2_. Following transfection, lentivirus particles were produced and collected in serum-free Opti-MEM1 (Thermo Fisher Scientific) for up to 48 h. Culture medium containing lentiviral particles was collected, filtered through a 0.45 μM Minisart filter (Sartorius), and concentrated by centrifugation at 4°C using a VIVASPIN 20 concentration column (Sartorius). Viral particles were stored at −80°C.

### Generation of Cell Lines

Lentiviral transduction was used to obtain SUPT1 and P12 Ichikawa T-ALL cell lines containing a variety of expression vectors. For this, viral batches were serially diluted in 96-well plates in a total volume of 50 μl of Opti-MEM1 medium, and 50,000 cells were added in Advanced RPMI 1640 medium (Thermo Fisher Scientific) supplemented with 2% heat-inactivated FCS, 1× Glutamax, 1% penicillin/streptomycin, and 0.25 μg/ml Fungizone. Cells were incubated at room temperature for 30 min on a gently shaking platform, and further incubated for several hours in a humidified incubator at 37°C and 5% CO_2_. For overnight incubation, FCS was added to a final concentration of 10%, after which the medium was refreshed. The transduction efficiency was determined 4 d later. To avoid multiple integrations per cell, only cells that were transduced by optimal virus concentrations (maximal transduction rate of 50%) were further cultured in bulk and enriched by selection in medium containing 1–2 μg/ml puromycin. The bulk-selected cells were used for subsequent experiments.

### Cytotoxicity Assays

Bulk transduced lines were maintained in culture medium at a concentration of 0.25–1.5 × 10^6^ cells/ml and refreshed twice weekly. To induce expression from the lentiviral vector, cells were grown in the presence of 0.5 mg/ml doxycycline prior to the cytotoxicity testing. Cytotoxicity was tested in the presence of a single concentration or a serial dilution of a variety of drugs or inhibitors as indicated: prednisolone (15 mg/ml–0.007 μg/ml), *L*-asparaginase (100–0.032 IU/ml), vincristine (2.5–0.10 ng/ml), 2 μM ruxolitinib (JAK1 inhibitor, Selleck Chem #S1378), 2 μM MK2206 (AKT inhibitor, Selleck Chem #S1078), 10 μM CI1040 (MEK inhibitor, Axon Medchem #Axon 1368), and 2 μM CAS 667463-62-9 (GSK3 inhibitor IX, Santa Cruz Biotechnology #sc-202634). Cells were incubated for 72 or 96 h in a humidified incubator at 37°C and 5% CO_2_ as specified in figure legends. Viable cell numbers were counted based on forward- and side-scatter parameters using flow cytometry (MACSQuant, Miltenyi Biotec), and data were analyzed using FlowJo software v10 (Treestar). Percent of viable cells after drug exposure was normalized to the percentage of viable untreated control cells, whereas percent of viable cells after exposure to a drug in combination with an inhibitor was normalized to the viability of inhibitor-treated control cells. The selected concentration of the inhibitors was such that the viability of inhibitor-treated control cells deviated not more than 20% compared to the viability of untreated control cells. Graphs were made using Graphpad PRISM 6 software.

### Western Blot Analysis and Antibodies

Primary antibodies used for Western blot detection of proteins were obtained from Cell Signaling Technology, unless specified otherwise: phospho-AKT S473 (#9271), phospho-JAK1 (#3331), phospho-STAT5 (#9351), phospho-MEK1/2 (#9154), phospho-ERK1/2 (#4370), phospho-mTOR S2448 (#2971), phospho-p70S6 Kinase T421/S424 (#9204), BAD (Abcam #AB32455), phospho-BAD S136 (#4366), BIM (Abcam #AB15184), phospho-BIM S55 (#4550), phospho-BIM S69 (#4581), phospho-CREB S133 (#9198), phospho-ATF1 (#9198), BCL2 (Santa Cruz Biotechnology #sc-130308), BCLXL (#2764), MCL1 (Sigma #HPA008455), PUMA (Abcam #AB33906), cMYB (#12319), phospho-IKKab S176/S177 (#2978), phospho-IKKab S176/S180 (#2697), GSK3AB (#5676), phospho-GSK3AB S21/9 (#9331), phospho-p38/MAPK T180/Y182 (#4511), GCR (Santa Cruz Biotechnology #sc-1003), phospho-GCR S211 (Abcam #AB3579), DYKDDDDK Tag Antibody (#2368), CD127 (IL7R) (R&D Systems #MAB306), RAS (Millipore #05–516), and β-actin (Abcam #AB6276). Following staining of Western blots using IRDye fluorescent secondary antibodies, fluorescent intensities were scanned and quantified using an Odyssey imager (LI-COR) and normalized to β-actin expression levels.

### Inhibitor Testing and Curve Shift Synergy Experiments on Primary T-ALL Patient Samples

Single drug or inhibitor cytotoxicity assays were carried out as described previously in 384-well plates for 72 h [33]. Briefly, 10,000 primary T-ALL patient cells from viably frozen stocks were plated in 45 μl of culture medium (Advanced RPMI 1640 medium [Thermo Fisher Scientific] containing 20% heat-inactivated fetal bovine serum, 1× Glutamax, 1% penicillin/streptomycin, 25 μg/ml gentamycin, and 0.25 μg/ml Fungizone) 2 h before drugs or inhibitors were added. To determine the IC50, each drug or inhibitor was dissolved in 100% DMSO in approximately 10,000-fold the estimated IC50 concentration, diluted in a nine-point dilution series in duplicate in DMSO, which was then 100-fold diluted in HEPES buffer (pH = 7.4). For the assay, 5 μl of each single drug or compound dilution was added to the preplated cells in a final concentration that ranged from 1 nM to 10,000 nM for all compounds in 0.1% DMSO. As an indirect measure of total cell viability after 72 h of incubation, the intracellular ATP content was measured. The plates were cooled to room temperature, and the cells were incubated with 25 μl of ATPlite 1step solution (PerkinElmer) per well. Luminescence was recorded on an Envision multimode reader (PerkinElmer), and results were normalized to the measurements of control wells containing only 0.1% DMSO. IC50 values were fitted manually by nonlinear regression using XLfit5 software (IDBS). Maximum and minimum signals were locked, where appropriate, to obtain the best fit as indicated by the F-test as implemented in XLfit5. If IC50 values did not fall within the tested concentration range, drugs or inhibitors were retested after further dilution. For curve shift synergy experiments of drug–inhibitor combinations, inhibitor stocks were diluted in DMSO on the day of the experiment to concentrations of 10,000 times their IC50 values, as determined in the single agent experiments. Single drug or inhibitor dilutions as well as drug–inhibitor combination mixtures in 1:1, 4:1, and 1:4 ratios were prepared, and diluted in DMSO into seven-point dose–response series in duplicate. Further dilution and addition to the cells was performed as described above. Because of the IC50 matching, all stock solutions and mixtures would be equipotent in the absence of synergy or antagonism. For the single drug or inhibitor experiments, final assay concentrations were between 10 and 0.01 times their IC50 values. For single drug or inhibitor curves, IC50 values were fitted on the percent-effect data by nonlinear regression using XLfit5. To correct for interassay variation, the IC50 values of the single agents in the synergy experiment were used to calculate the synergy for the drug–inhibitor combination experiments. The combination index (CI) as a quantitative measure of synergy for drug–inhibitor combinations [34] was calculated as described previously [35]. CI values lower than 1.0 indicate synergy of drug–inhibitor combinations; CI values lower than 0.3 indicate strong synergy. CI values higher than 1.5 indicate antagonistic effects of drug–inhibitor combinations.

## Results

### Whole Genome and Targeted Exome Sequencing of Pediatric T-ALL Patient Samples

To determine the mutational landscape of pediatric T-ALL patients, we used an integrated approach combining mutation data, copy number data, and clinical information obtained from 82 pediatric T-ALL patients (Fig 1; S1 Table). First, we performed WGS on paired diagnostic–remission samples from 13 patients at Complete Genomics [21]. These patients covered all known genetic subtypes including two early thymic progenitor ALL (ETP-ALL) cases; cases harboring *TLX3* (five cases), *TLX1* (one case), *NKX2-1* (one case), *HOXA* (one case), *KTM2A/MLL* (one ETP-ALL case), or *TAL1* (one case) chromosomal rearrangements; and cases for which driving oncogenic events remain unknown (three patients, including one ETP-ALL case) [32]. Extensive filtering (S1 Fig) and first quality parameter optimization was performed that was validated by PCR–Sanger sequencing (S2 Fig). We established the optimal quality parameter thresholds for WGS analysis to robustly call high-confidence mutations as being 0.1 and 100 for SS and TS, respectively. We further confirmed these thresholds by TES using the Illumina HiSeq 2000 platform of 410 genes from the diagnostic samples of these same 13 T-ALL patients (S1 and S2 Tables). These genes included 137 genes containing 178 high-confidence mutations (85 SNVs and 93 INDELs) and 273 genes containing 377 predicted low-confidence mutations (139 SNVs and 238 INDELs). We confirmed 74 out of 85 (87%) high-confidence SNVs and two out of 93 (2%) high-confidence INDEL mutations, in contrast to seven out of 139 (5%) low-confidence SNVs and zero out of 238 low-confidence INDEL mutations. These findings indicate that the chosen quality parameter thresholds for WGS were suitable to identify high-confidence mutations. Moreover, the overlap between WGS and TES results was further used to optimize the TES quality parameter threshold setting (S3 Fig).

**Fig 2:**
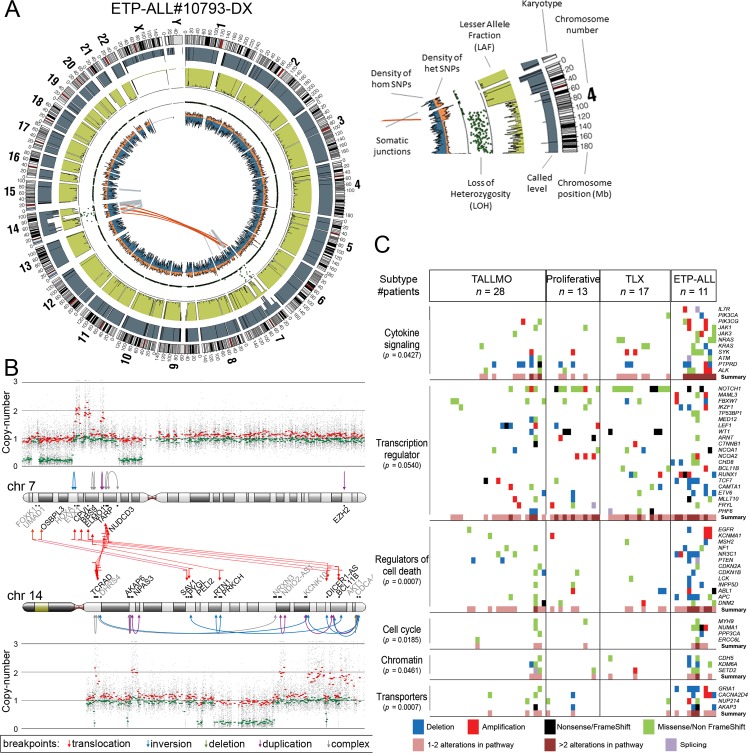
Whole genome and targeted exome sequencing results for pediatric T-ALL patients at diagnosis. (A and B) Visualization of chromosomal breakpoint junctions in diagnostic leukemia cells of ETP-ALL patient #10793. (A) Circos plot of somatic structural variations as detected in the diagnostic sample (Dx) along with SNV densities (1-Mb window) and predicted LOH data as explained in the accompanying legend. Interchromosomal junctions are displayed as red lines, and intrachromosomal junctions are displayed as grey lines. (B) Allele-specific copy number variations as determined by Affymetric SNP Array analysis and multiple WGS-predicted chromosomal breakpoints as a consequence of chromothripsis affecting Chromosomes 7 and 14 are displayed. In the copy number plots, black dots indicate the unsmoothed allele-specific copy numbers. Red dots are the smoothed maximal allele-specific copy numbers using a sliding window of 30 SNP probes, while the green dots reflect the smoothed minimal allele-specific copy numbers. Chromosomal breakpoint junctions are displayed for translocations (red arrows), inversions (blue arrows), deletions (green arrows), duplications (purple arrows), and complex rearrangements (grey arrows). Affected (in black) and flanking (in grey) genes for the interchromosomal translocations are indicated. (C) Overview of most deregulated cellular processes among 127 genes carrying mutations/aberrations in the diagnostic material of two or more patients in the expansion cohort of 69 T-ALL patients. The number of patients with each T-ALL subtype is indicated. The *p*-value for each process represents the significance level for enrichment of mutations/aberrations that affect this pathway in ETP-ALL patients compared to other T-ALL subtypes, and was calculated by two-sided Fisher’s exact test. See also S4 Table. ETP-ALL, early thymic progenitor acute lymphoblastic leukemia; LOH, loss of heterozygosity; SNV, single nucleotide variant; T-ALL, T cell acute lymphoblastic leukemia.

In addition to mutations, we identified by WGS 185 somatic intrachromosomal breakpoint junctions (average 14 per patient, range 5–25) and 40 interchromosomal breakpoint junctions (average three per patient, range 2–11) that represented 183 predicted rearrangements including 78 deletions, 28 duplications, 16 inversions, 16 translocations, and 45 complex rearrangements in these 13 patients (S2 Table). In all, 182 out of 225 chromosomal junctions had evidence for inclusion of random nucleotides (average 18 nucleotides, range 1–221) that point to the involvement of the RAG recombination machinery. This presence of random nucleotides was also the case for most chromosomal junctions identified in the two ETP-ALL patients, both of whom arrested at early thymic progenitor stages that precede RAG-mediated T cell receptor gene recombination events. Breakpoints for known driving oncogenic rearrangements as predicted using fluorescence in situ hybridization or real-time quantitative PCR were identified in ten out of 13 T-ALL patients. We could not pinpoint any specific oncogene-driving event from the various rearrangements identified in two out of three T-ALL cases for which driving oncogenic rearrangements were unknown (#9255 and #9343). One ETP-ALL patient (#10793) had evidence for chromothripsis in Chromosomes 7 and 14 (Fig 2A). Breakpoints for deletions, insertions, duplications, complex rearrangements, and translocations were identified in both chromosomes that flank areas of allelic losses or gains according to SNP-based microarray analysis (Fig 2B). Multiple breakpoints were identified close to the *HOXA* gene cluster. Multiple clustered breakpoints frequently affected *TCRAD* and *BCL11B* loci and pointed to a rather organized form of chromothripsis in this particular patient rather than random reassembly of disintegrated chromosomal segments as frequently observed in patients with chromothripsis [36].

### The Mutational Landscape of T-ALL and Associations with Steroid Resistance

We then expanded our mutation analysis by performing TES on a cohort of diagnostic samples from 69 well-characterized pediatric T-ALL patients (Fig 1; S1 Table) [37]. In total, 254 genes were sequenced at a mean coverage of 561×, including the 144 validated genes identified in the discovery cohort and 110 additional selected genes that are frequently mutated in ALL (S3 Table) [24,25]. Following stringent filtering (S4 Fig), 401 mutations were identified in 151 genes (S3 Table). These mutations were then integrated with aCGH copy number data, which were available for 53 out of 69 patients (S3 Table). The median number of genes harboring mutations and/or copy number changes was six per patient (range 0–51). ETP-ALL patients had more aberrations than other subtypes (*p <* 0.001, median 12, range 5–51) [32,38,39], while TALLMO patients had fewer aberrations (*p =* 0.003, median 4, range 0–42). The median number of aberrations in TLX and proliferative subtypes was 8.5 (range 4–10) and 6 (range 3–27), respectively. Sixty-six of the mutated genes have been previously identified in T-ALL, and 83 mutated genes have been previously found in other types of leukemia or nonhematopoietic tumors. Two genes, *RABL6* (a GTP-binding member of the Ras superfamily of small GTPases) and *IGHV3-64*, have not previously been reported in mutated form in human cancer. Mutations mostly affected genes that are involved in cytokine signaling, transcription regulation, cell death, cell cycle, or chromatin modification or that encode transporters (Fig 2C; S4 Table).

**Fig 3:**
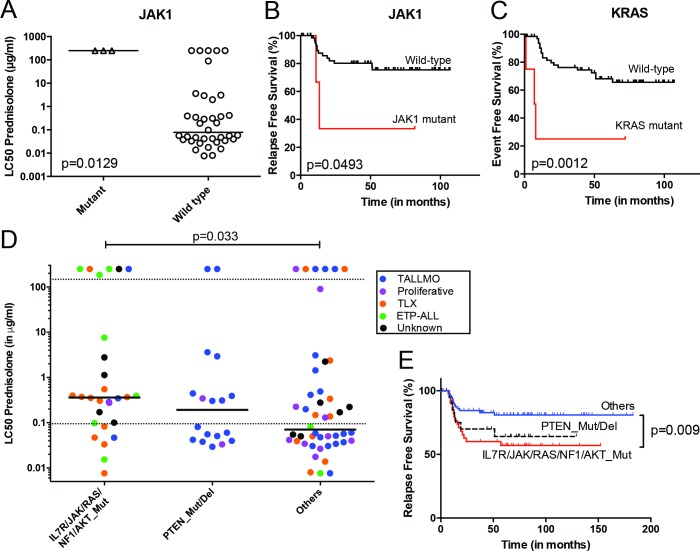
Mutations/aberrations affecting the IL7R signaling pathway in pediatric T-ALL patients at diagnosis predict diminished steroid response and poor outcome. Mutations in (A and B) *JAK1* or (C) *KRAS* detected by TES in diagnostic samples from 69 T-ALL patients are associated with diminished steroid response and/or poor survival. IL7R signaling mutations in diagnostic samples from 146 T-ALL patients are associated with reduced (D) in vitro steroid sensitivity and (E) relapse-free survival. Patients harboring *NR3C1* deletion as a consequence of a chromosomal 5q deletion were excluded from these analyses. See also S6 Table. ETP-ALL, early thymic progenitor acute lymphoblastic leukemia; T-ALL, T cell acute lymphoblastic leukemia; TES, targeted exome sequencing.

The mutation/aberration statuses of the 151 genes were then correlated with clinical and biological parameters of these 69 patients: in vitro drug response, T-ALL subtype, and outcome. Several mutations and aberrations were associated with poor survival or drug resistance (S5 Table). Interestingly, mutations in IL7R signaling molecules—including *JAK1* and *KRAS*—correlated with prednisolone resistance and reduced survival (Fig 3A–3C). To validate these associations, we expanded our mutation analyses by PCR–Sanger sequencing to the *IL7R*, *JAK1*, *JAK3*, *NF1*, *NRAS*, *KRAS*, and *AKT* genes in the diagnostic samples of these 69 patients and 77 additional T-ALL patients. Mutations were identified in 47 out of 146 patients (32%) and were associated with the ETP-ALL and TLX subtypes (*p <* 0.001; see S1 and S6 Tables for details). In vitro prednisolone response data on these diagnostic samples were available for 97 patients, and in 28 out of 97 cases IL7R signaling mutations were associated with steroid resistance (*p =* 0.033; Fig 3D; S6 Table). These patients also had a significantly worse outcome than patients lacking these mutations (*p =* 0.009; Fig 3E). Inactivating events in PTEN in 18 out of 97 patients are associated with the TALLMO subtype [20,40], and these patients had prednisolone sensitivity levels that were similar to those of patients lacking IL7R signaling or *PTEN* mutations. These data therefore suggest a potential relationship between IL7R signaling mutations and steroid resistance.

### Mutations in IL7R Signaling Molecules Confer Steroid Resistance

**Fig 4:**
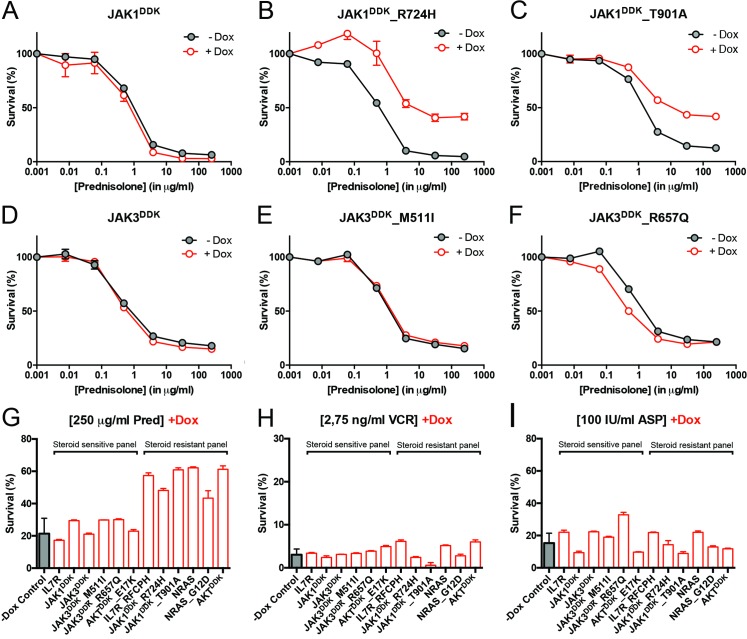
Activating IL7R signaling mutations can confer resistance to steroid treatment. (A–F) Steroid response curves (from triplicate experiments ± standard deviation) for SUPT1 cells that express (A) JAK1, (B) JAK1^R724H^, (C) JAK1^T901A^, (D) JAK3, (E) JAK3^M511I^, or (F) JAK3^R657Q^ from doxycycline-inducible lentiviral expression constructs. Steroid response curves are shown for induced (+Dox) and non-induced (−Dox) cells that have been exposed to serial dilutions of prednisolone for 72 h. (G–I) Mean survival of SUPT1 cells (triplicate experiments ± standard deviation) expressing wild-type or mutant IL7R signaling molecules (+Dox: open red bars) following a 72-h exposure to (G) prednisolone (Pred), (H) vincristine (VCR), or (I) *L*-asparaginase (ASP). Black bars represent the mean survival of all SUPT1 lines under non-induced conditions following exposure to prednisolone, vincristine, or *L*-asparaginase (−Dox control). The steroid-sensitive panel refers to SUPT1 lines that retain a similarly sensitive steroid response following expression of IL7R, JAK1, JAK3, JAK3^M511I^, JAK3^R657Q^, or AKT^E17K^ compared to non-induced control conditions. The steroid-resistant panel refers to lines that acquire steroid resistance following expression of IL7R^RFCPH^, JAK1^R724H^, JAK1^T901A^, NRAS, NRAS^G12D^, or AKT. See also S5 Fig.

To functionally explore whether IL7R signaling mutations may drive steroid resistance, we tested whether expression of mutant IL7R signaling molecules—versus their wild-type counterparts—can confer steroid resistance in two steroid-sensitive T-ALL lines, namely SUPT1 and P12 Ichikawa. All mutant signaling molecules expressed from doxycycline-inducible expression constructs—including IL7R^RFCPH^, JAK1^R724H^, JAK1^T901A^, JAK3^M511I^, JAK3^R657Q^, NRAS^G12D^, and AKT^E17K^—were able to trigger IL3-independent growth in Ba/F3 cells, in contrast to their wild-type isoforms, confirming that these mutations can transform cells [41]. In SUPT1 and P12 Ichikawa T-ALL backgrounds, expression of the cysteine mutant IL7R^RFCPH^ conferred steroid resistance, while the wild-type IL7R and the non-cysteine IL7R^GPSL^ mutant receptors did not (S5 Fig). Both mutant JAK1 molecules—but not wild-type JAK1—conferred steroid resistance (Fig 4A–4C). Surprisingly, JAK3 mutations did not confer resistance (Fig 4E and 4F), while expression of wild-type NRAS, NRAS^G12D^, and wild-type AKT strongly conferred resistance to steroids (Figs 4G and S5). Although AKT^E17K^ behaves as an activating mutant [42] that supports IL3-independent growth in Ba/F3 cells [41], it did not confer steroid resistance in SUPT1 or P12 Ichikawa cells. We therefore denoted bulk lines that expressed IL7R^RFCPH^, JAK1^R724H^, JAK1^T901A^, NRAS^G12D^, or wild-type NRAS or AKT as the “steroid-resistant panel,” whereas lines that expressed wild-type IL7R, JAK1, or JAK3 or mutant JAK3^M511I^, JAK3^R657Q^, or AKT^E17K^ as the “steroid-sensitive panel.” The expression of particular (mutant) IL7R signaling molecules specifically affected steroid response since all lines displayed comparable sensitivities to vincristine and *L*-asparaginase treatments (Figs 4H, 4I and S5).

### IL7R Signaling Mutations Do Not Affect the Function of NR3C1

We next investigated whether the expression of IL7R signaling molecules that give rise to steroid resistance affects nuclear shuttling of the steroid receptor NR3C1, rendering it incapable of activating downstream target genes upon steroid exposure. We found no difference among steroid-resistant and steroid-sensitive lines in their abilities to activate NR3C1 target genes following steroid exposure, including *NR3C1*, *TSC22D3/GILZ*, *BCC3/PUMA*, *KLF13*, *BCL2L11/BIM* and *FKBP5* (S6 Fig). Therefore, the steroid resistance provoked by expression of mutant IL7R, JAK1, or NRAS molecules, or wild-type NRAS or AKT, is independent of the NR3C1 response following steroid exposure.

### Crosstalk between the NR3C1 and IL7R Signaling Pathways

**Fig 5:**
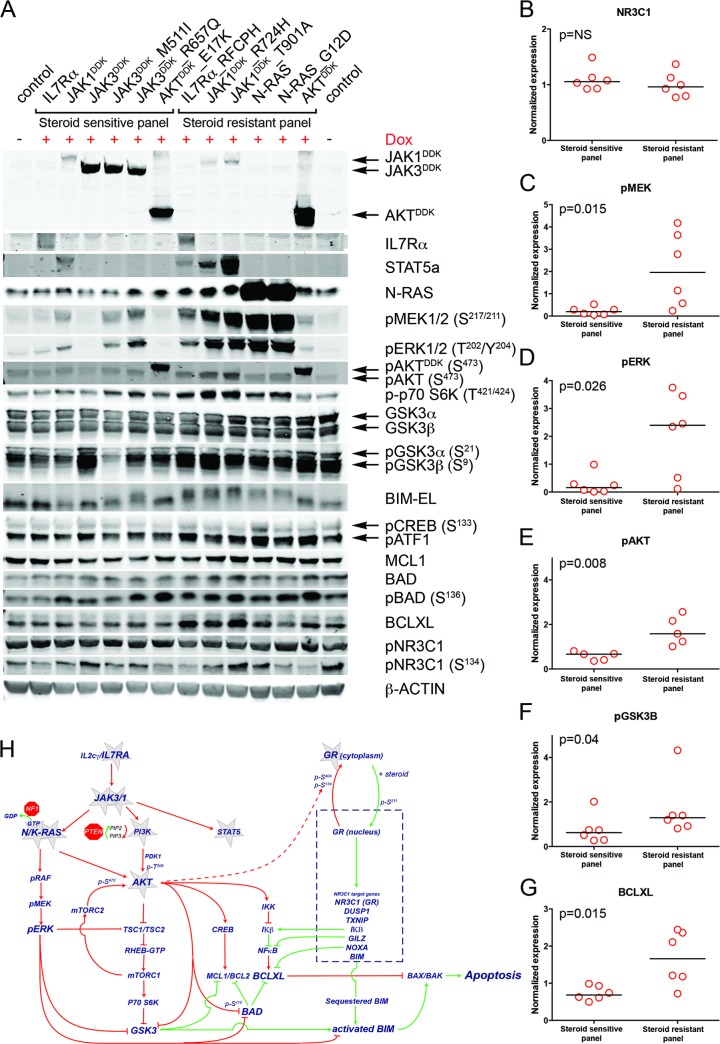
Steroid resistance induced by wild-type or mutant IL7R signaling molecules is associated with activation of MEK-ERK and/or AKT. (A) Western blot results for total and/or phosphorylated levels of IL7R signaling molecules following doxycycline induction (+Dox) of wild-type or mutant forms of IL7R, JAK1, JAK3, NRAS, or AKT molecules in SUPT1 cells. The steroid-sensitive and -resistant panels are indicated. Cellular lysate of parental SUPT1 cells was used as a control. (B–G) β-actin-normalized protein concentrations for (B) NR3C1, (C) pMEK, (D) pERK, (E) pAKT, (F) pGSK3B, and (G) BCLXL in doxycycline-induced steroid-sensitive and -resistant SUPT1 lines. Significance levels were calculated using the Mann-Whitney *U* test. In (E), phospho-AKT levels are shown for all lines except for lines induced to express construct-driven AKT and AKT^E17K^. (H) Schematic overview of crosstalk between the proapoptotic NR3C1 response following steroid exposure and activation of MEK-ERK and AKT pathways downstream of IL7R signaling mutations. Green vectors indicate molecules that drive a proapoptotic, steroid-sensitive response, whereas red vectors indicate molecules that drive an antiapoptotic, steroid-resistant response. See also S7 Fig.

To examine the underlying mechanism that confers steroid resistance, we performed Western blot analyses on steroid-resistant and steroid-sensitive lines to measure the activities of the IL7R signaling and downstream pathways (Figs 5A and S7). Steroid-sensitive and -resistant lines had equal concentrations of total NR3C1 and equal serine 134 phosphorylation (Fig 5A and 5B). In contrast to steroid-sensitive lines, steroid-resistant lines had higher activation levels of the RAS-MEK-ERK and AKT pathways (Fig 5C–5E). Steroid-resistant lines also displayed higher levels of phosphorylated (activated) p70-S6K and (inactivated) GSK3B (Fig 5F), and higher activation of the CREB and NFκB pathways downstream of AKT, which resulted in significantly higher concentrations of antiapoptotic MCL1 and BCLXL (Fig 5G). Thus, robust IL7R pathway activation in steroid-resistant lines triggered a strong survival response, which may have overridden the proapoptotic NR3C1 response (Fig 5H). Although BIM is essential for steroid-induced apoptosis [43–46], total BIM concentrations were comparable among steroid-sensitive and -resistant lines. All steroid-resistant lines had higher levels of phosphorylated BIM except for the steroid-resistant AKT line (Fig 5A). Consistent with this, GSK3B, an important regulator of proapoptotic BIM [47,48], was strongly inactivated in all these resistant lines.

### IL7R Signaling Inhibitors Revert Steroid Resistance in T-ALL

**Fig 6:**
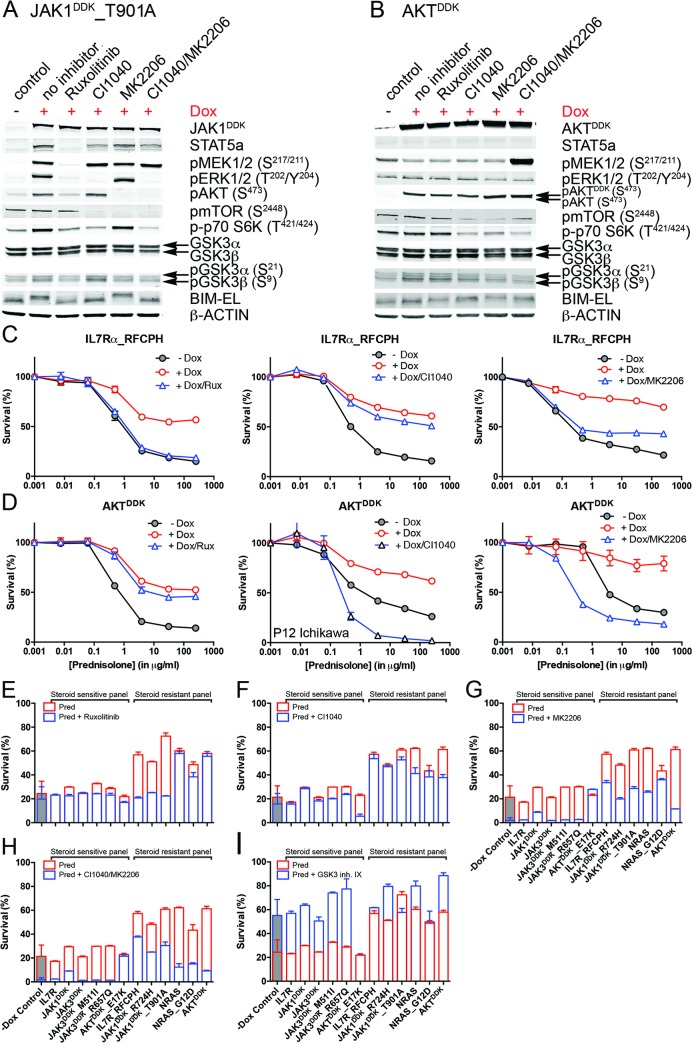
Reversal of steroid resistance by IL7R signaling inhibitors. Activity status of IL7R signaling molecules by Western blot analysis in SUPT1 cells expressing (A) JAK1^T901A^ or (B) wild-type AKT that are exposed to ruxolitinib (2 μM), CI1040 (10 μM), MK2206 (2 μM), or the CI1040/MK2206 combination for 24 h compared to non-induced and doxycycline-induced controls. (C and D) SUPT1 or P12 Ichikawa (D, middle panel) T cell acute lymphoblastic leukemia cell response curves following 72-h exposure to serial dilutions of prednisolone (triplicate experiments ± standard deviation) without (−Dox, grey circles) or with (+Dox, open red circles) induction of (C) IL7R^RFCPH^ or (D) AKT. The effect of 2 μM ruxolitinib (left panels), 10 μM CI1040 (middle panels), and 2 μM MK2206 (right panels) on the steroid response under doxycycline-induced conditions are shown (open blue triangles). (E–I) Mean survival (triplicate experiments ± standard deviation) of SUPT1 lines expressing wild-type or mutant IL7R signaling molecules following 72-h treatment with prednisolone (250 μg/ml) in the absence (open red bars) or presence (open blue bars) of (E) ruxolitinib, (F) CI1040, (G) MK2206, (H) the combination of CI1040/MK2206, or (I) GSK3 inhibitor IX. Steroid-sensitive and -resistant panels are indicated. For each experiment, the mean survival for all non-induced (−Dox) SUPT1 lines that are exposed to prednisolone (red, grey-filled bar) or prednisolone plus inhibitor (blue, grey-filled bar) is shown as a control. See also S8 Fig. Dox, doxycycline; inh., inhibitor; Pred, prednisolone; Rux, ruxolitinib.

We then tested whether inhibitors of IL7R signaling can restore steroid sensitivity in steroid-resistant cells. Inhibitors of JAK1 (2 μM ruxolitinib), MEK (10 μM CI1040), and AKT (2 μM MK2206) were tested for their specificity in blocking IL7R signaling in steroid-resistant lines expressing IL7R^RFCPH^, JAK1^T901A^, AKT, or NRAS and their abilities to revert steroid resistance. Ruxolitinib blocked IL7R downstream signaling in both IL7R^RFCPH^ and JAK1^T901A^ lines, but was ineffective in blocking signaling in AKT or NRAS lines, as expected (Figs 6A, 6B and S8). Consistently, ruxolitinib treatment increased steroid sensitivity in IL7R^RFCPH^ and mutant JAK1 lines (Fig 6C and 6E) but not in wild-type NRAS or AKT lines (Figs 6D, 6E and S8). The MEK inhibitor CI1040 blocked ERK activation in all four lines tested, reducing downstream activation of mTOR and p70-S6K. CI1040 treatment enhanced activation of GSK3B and caused a shift from phosphorylated to non-phosphorylated BIM in these lines, except for the AKT line. CI1040 treatment also resulted in elevated levels of active AKT, possibly due to a cellular feedback rescue mechanism. CI1040 partially restored steroid sensitivity in most steroid-resistant SUPT1 lines and was most effective in the wild-type NRAS and mutant NRAS^G12D^ lines (Figs 6C, 6D and 6F). CI1040 treatment efficiently enhanced steroid sensitivity in resistant P12 Ichikawa lines that expressed IL7R^RFCPH^ or wild-type NRAS or AKT (S8 Fig). CI1040 also increased steroid sensitivity in some steroid-sensitive lines (Figs 6F and S8). The AKT inhibitor MK2206 blocked AKT signaling and reduced phosphorylated levels of downstream mTOR and p70-S6K (Fig 6A and 6B). MK2206 restored the steroid sensitivity phenotype in all steroid-resistant lines (Figs 6C, 6D, 6G and S8). Like CI1040 treatment, MK2206 treatment also increased steroid sensitivity in steroid-sensitive lines, possibly by inhibiting endogenous AKT. Similar effects were observed for treatment with the MEK-AKT inhibitor combination (Fig 6H). In contrast, inhibitor IX, a blocker of GSK3B activation, provoked resistance in the majority of steroid-sensitive lines as well as some steroid-resistant lines (Fig 6I), providing further evidence that GSK3B is a key regulator of steroid responsiveness.

We then further tested whether clinically relevant inhibitors of the IL7R signaling pathway affected the prednisolone sensitivity of primary leukemia cells isolated from 11 pediatric T-ALL patients at diagnosis (Tables 1 and S1). Leukemia cells were incubated with serial dilutions of prednisolone or single inhibitors as well as their combinations at different ratios based on estimated IC50 concentrations. The level of synergism was determined by calculating the CI for serial dilutions of three drug–inhibitor combination mixtures (1:1; 1:4, and 4:1) at effective doses that were lethal for 50% (ED50) or 75% (ED75) of the leukemia cells (S9 Fig). Three out of 11 patients harbored *IL7R* mutations, whereas one patient had a *KRAS* mutation. Leukemic cells from four other patients did not harbor mutations in IL7R signaling components but had other mutations, whereas the mutation status of three additional patients was unknown (Table 1). These 11 patient samples differed in their responses to prednisolone based on their IC50 values or the percentage of total responding cells (Table 1), and patients #2911 and #10110 were refractory to steroid treatment. Surprisingly, in contrast to previous results on cell lines, the JAK inhibitor ruxolitinib had no effect on the prednisolone response in the eight T-ALL patient samples for which CI values could be calculated. Treatment with the MEK inhibitor AZD6244 or trametinib synergistically enhanced prednisolone responsiveness in 5/9 and 9/10 patient samples, respectively. Neither inhibitor enhanced steroid responsiveness in the leukemia cells from steroid-resistant patient #2911, whereas trametinib synergistically enhanced the prednisolone response in leukemia cells from steroid-resistant patient #2322. AKT inhibitor MK2206 synergistically enhanced prednisolone sensitivity in leukemia cells from 8/11 patients, while mTOR inhibitors AZD8055 and everolimus acted synergistically when combined with prednisolone in 10/11 and 8/8 patient samples, respectively, including both steroid-resistant patients. The PI3K inhibitor NVPBEZ235 synergistically enhanced prednisolone sensitivity in 10/11 patient samples.

**Table 1:** MEK and PI3K/AKT pathway inhibitors enhance the steroid response of primary T-ALL patient samples.

*Patient*	Chemotherapy		Inhibitor			CI ± SD		Synergy Level
	Drug	IC50 [nM] (Maximum Effect)	Compound	Target	IC50 [nM] (Maximum Effect)	ED50	ED75	
***T-ALL #9858 (TLX3***^***tr***^**, *p16/p15***^***DEL/DEL***^***; mutations in IL7R*, *NRAS*, *NOTCH1 [HD domain]*, *WT1*, *BCL11B*, *CDH9*, *STIL)***								
	Prednisolone	18 (80%)	Ruxolitinib	JAK1	205 (<20%)	1.40*	NA	No synergy
	Prednisolone	17 (77%)	AZD6244	MEK1/2	71 (76%)	**0.61 ± 0.04**	**0.13 ± 0.04**	**Synergy/strong synergy**
	Prednisolone	14 (73%)	Trametinib	MEK1/2	11 (79%)	0.86 ± 0.27	**0.26 ± 0.18**	**Strong synergy**
	Prednisolone	18 (74%)	MK2206	AKT	60 (27%)	0.98 ± 0.36	NA	No synergy
	Prednisolone	12 (74%)	AZD8055	mTOR	288 (74%)	**0.67 ± 0.14**	NA	**Synergy**
	Prednisolone	17 (75%)	Everolimus	mTOR	12,614 (<20%)	0.61*	<0.10*	**Synergy/strong Synergy**
	Prednisolone	14 (72%)	NVPBEZ235	mTOR/PI3K	322 (59%)	0.98 ± 0.19	NA	No synergy
***T-ALL #1570 (HOXA*, *mutations in IL7R*, *NOTCH1 [JM domain]*, *FREM2*, *RUNX1)***								
	Prednisolone	25 (65%)	Ruxolitinib	JAK1	58 (<20%)	1.49 ± 0.74	NA	No synergy
	Prednisolone	36 (73%)	AZD6244	MEK1/2	647 (40%)	0.57 ± 0.50	NA	No synergy
	Prednisolone	19 (53%)	Trametinib	MEK1/2	49 (24%)	**0.46 ± 0.16**	NA	**Synergy**
	Prednisolone	20 (63%)	MK2206	AKT	464 (39%)	1.12 ± 0.62	NA	No synergy
	Prednisolone	29 (82%)	AZD8055	mTOR	22 (<20%)	0.55*	NA	**Synergy**
	Prednisolone	21 (62%)	Everolimus	mTOR	72 (<20%)	0.85*	NA	**Synergy**
	Prednisolone	57 (79%)	NVPBEZ235	mTOR/PI3K	728 (69%)	**0.54 ± 0.06**	**0.39 ± 0.42**	**Synergy**
***T-ALL #2911 (HOXA***^***r***^ ***[Inv(7)]*, *p16/p15***^***DEL/DEL***^***; mutations in IL7R*, *NOTCH1 [PEST domain]*, *WT1*, *ZNF717)***								
	Prednisolone	9,007 (<20%)	Ruxolitinib	JAK1	5,323 (59%)	1.65 ± 0.28	NA	No synergy
	Prednisolone	425 (<20%)	AZD6244	MEK1/2	246 (58%)	0.61 ± 0.59	NA	No synergy
	Prednisolone	90 (<20%)	Trametinib	MEK1/2	81 (75%)	0.90 ± 0.25	NA	No synergy
	Prednisolone	154 (<20%)	MK2206	AKT	239 (51%)	**0.22 ± 0.07**	NA	**Strong synergy**
	Prednisolone	104 (<20%)	AZD8055	mTOR	251 (64%)	**0.44 ± 0.09**	NA	**Synergy**
	Prednisolone	560 (<20%)	Everolimus	mTOR	68 (51%)	NA	NA	NA
	Prednisolone	>10,000 (<20%)	NVPBEZ235	mTOR/PI3K	362 (71%)	**0.30 ± 0.05**	NA	**Synergy**
***ETP-ALL #491 (MEF2C***^***tr***^**, *NR3C1***^***DEL***^**, *mutations in KRAS*, *NOTCH1 [HD/PEST domains]*, *ADAMTS5*, *ALK*, *CDH8*, *DNM2*, *FAM171A1*, *NCOA2*, *NOTCH1*, *NOTCH3*, *NOTCH4*, *NUMA1*, *PTCH1*, *SETD2*, *SYK*, *TP53BP1)***								
	Prednisolone	120 (87%)	Ruxolitinib	JAK1	ND (<20%)	NA	NA	NA
	Prednisolone	80 (70%)	AZD6244	MEK1/2	141 (41%)	**0.29 ± 0.13**	<0.10*	**Strong synergy**
	Prednisolone	105 (67%)	Trametinib	MEK1/2	13 (24%)	**0.33 ± 0.17**	NA	**Synergy**
	Prednisolone	123 (61%)	MK2206	AKT	ND (<20%)	0.70*	NA	**Synergy**
	Prednisolone	78 (82%)	AZD8055	mTOR	ND (<20%)	1.41*	NA	No synergy
	Prednisolone	61 (76%)	Everolimus	mTOR	ND (<20%)	0.79*	0.41*	**Synergy**
	Prednisolone	95 (62%)	NVPBEZ235	mTOR/PI3K	ND (<20%)	0.60*	NA	**Synergy**
***T-ALL #1032 (TALLMO-like*, *p16/p15***^***GL/DEL***^***; mutations in PTEN*, *KIF14*, *NCOA1*, *TET2)***								
	Prednisolone	38 (91%)	Ruxolitinib	JAK1	ND (<20%)	1.12*	1.08*	No synergy
	Prednisolone	41 (89%)	AZD6244	MEK1/2	259 (18%)	**0.70 ± 0.08**	**0.88 ± 0.05**	**Synergy**
	Prednisolone	40 (89%)	Trametinib	MEK1/2	50 (25%)	**0.65 ± 0.05**	**0.79 ± 0.16**	**Synergy**
	Prednisolone	37 (87%)	MK2206	AKT	520 (81%)	0.96 ± 0.09	**0.70 ± 0.11**	**Synergy**
	Prednisolone	39 (91%)	AZD8055	mTOR	61 (68%)	0.98 ± 0.13	**0.58 ± 0.21**	**Synergy**
	Prednisolone	36 (89%)	Everolimus	mTOR	11 (51%)	**0.46 ± 0.01**	**0.84 ± 0.08**	**Synergy**
	Prednisolone	38 (89%)	NVPBEZ235	mTOR/PI3K	134 (64%)	1.24 ± 0.04	**0.63 ± 0.32**	**Synergy**
***T-ALL #1815 (TALLMO-like*, *MYB***^***dup***^**, *P16***^***DEL/DEL***^***; mutations in NOTCH1 [JM domain]*, *CACNA1E*, *NR3C1*, *ZNF717)***								
	Prednisolone	108 (52%)	Ruxolitinib	JAK1	ND (<20%)	1.39*	NA	No synergy
	Prednisolone	116 (59%)	AZD6244	MEK1/2	151 (53%)	**0.13 ± 0.03**	**<0.10**	**Strong synergy**
	Prednisolone	141 (50%)	Trametinib	MEK1/2	23 (53%)	**0.16 ± 0.07**	<0.10*	**Strong synergy**
	Prednisolone	119 (52%)	MK2206	AKT	165 (50%)	**0.19 ± 0.07**	<0.10*	**Strong synergy**
	Prednisolone	173 (68%)	AZD8055	mTOR	52 (58%)	**0.41 ± 0.06**	**<0.10**	**Synergy/strong synergy**
	Prednisolone	120 (59%)	Everolimus	mTOR	11 (36%)	**0.25 ± 0.06**	NA	**Strong synergy**
	Prednisolone	138 (53%)	NVPBEZ235	mTOR/PI3K	196 (57%)	**0.79 ± 0.05**	**<0.10**	**Synergy/strong synergy**
***T-ALL #2322 (LMO2***^***tr***^**, *p16***^***DEL/DEL***^***; mutations in NOTCH1 [HD domain]*, *CACNA2D4*, *KCNMA1*, *STAM)***								
	Prednisolone	122 (27%)	Ruxolitinib	JAK1	ND (<20%)	NA	NA	NA
	Prednisolone	49 (28%)	AZD6244	MEK1/2	223 (48%)	NA	NA	NA
	Prednisolone	ND (<20%)	Trametinib	MEK1/2	87 (465)	**<0.10**	NA	**Strong synergy**
	Prednisolone	86 (13%)	MK2206	AKT	504 (58%)	**0.10 ± 0.02**	<0.10*	**Strong synergy**
	Prednisolone	107 (30%)	AZD8055	mTOR	90 (57%)	**0.46 ± 0.15**	**<0.10**	**Synergy/strong synergy**
	Prednisolone	ND	Everolimus	mTOR	ND	ND	ND	ND
	Prednisolone	73 (21%)	NVPBEZ235	mTOR/PI3K	337 (92%)	**0.52 ± 0.14**	**0.59 ± 0.29**	**Synergy**
***T-ALL #10110 (LMO2***^***r***^ ***[del11(p12;p13)]*, *p16/p15***^***DEL/DEL***^***; mutations in NOTCH1 [HD domain]*, *FBXW7*, *ACAN*, *ATRX*, *DNM2*, *RPL10)***								
	Prednisolone	28 (84%)	Ruxolitinib	JAK1	122 (79%)	1.12 ± 0.11	0.82 ± 0.25	No synergy
	Prednisolone	ND	AZD6244	MEK1/2	ND	ND	ND	ND
	Prednisolone	ND	Trametinib	MEK1/2	ND	ND	ND	ND
	Prednisolone	39 (67%)	MK2206	AKT	4,035 (72%)	0.95 ± 0.13	<0.10*	**Strong synergy**
	Prednisolone	40 (86%)	AZD8055	mTOR	84 (72%)	0.96 ± 0.01	**0.65 ± 0.25**	**Synergy**
	Prednisolone	43 (92%)	Everolimus	mTOR	14 (54%)	**0.70 ± 0.17**	**0.53 ± 0.16**	**Synergy**
	Prednisolone	35 (74%)	NVPBEZ235	mTOR/PI3K	186 (50%)	1.21 ± 0.17	**<0.10**	**Strong synergy**
***T-ALL #3594 (TLX3***^***tr***^***)***								
	Prednisolone	46 (55%)	Ruxolitinib	JAK1	ND (<20%)	1.55*	NA	No synergy
	Prednisolone	63 (65%)	AZD6244	MEK1/2	79 (72%)	**0.38 ± 0.03**	<0.10*	**Synergy/strong synergy**
	Prednisolone	60 (62%)	Trametinib	MEK1/2	16 (76%)	**0.46 ± 0.12**	**<0.10**	**Synergy/strong synergy**
	Prednisolone	45 (52%)	MK2206	AKT	2,029 (100%)	**0.78 ± 0.09**	**0.32 ± 0.20**	**Synergy**
	Prednisolone	48 (63%)	AZD8055	mTOR	142 (60%)	0.78 ± 0.31	**<0.10**	**Strong synergy**
	Prednisolone	62 (66)	Everolimus	mTOR	ND (<20%)	0.41*	<0.10*	**Strong synergy**
	Prednisolone	36 (49%)	NVPBEZ235	mTOR/PI3K	326 (69%)	0.64 ± 0.43	**<0.10**	**Strong synergy**
***T-ALL #4992 (SIL-TAL1)***								
	Prednisolone	60 (94%)	Ruxolitinib	JAK1	2,384 (38%)	0.85 ± 0.18	1.00 ± 0.11	No synergy
	Prednisolone	50 (90%)	AZD6244	MEK1/2	99 (45%)	**0.43 ± 0.20**	**0.44 ± 0.14**	**Synergy**
	Prednisolone	63 (93%)	Trametinib	MEK1/2	61 (42%)	**0.49 ± 0.15**	**0.54 ± 0.13**	**Synergy**
	Prednisolone	112 (87%)	MK2206	AKT	723 (54%)	**0.55 ± 0.13**	**0.49 ± 0.15**	**Synergy**
	Prednisolone	44 (96%)	AZD8055	mTOR	14 (57%)	1.02 ± 0.13	**0.60 ± 0.17**	**Synergy**
	Prednisolone	38 (91%)	Everolimus	mTOR	2 (50%)	**0.34 ± 0.07**	**0.67 ± 0.05**	**Synergy**
	Prednisolone	103 (84%)	NVPBEZ235	mTOR/PI3K	496 (52%)	**0.61 ± 0.22**	**0.44 ± 0.19**	**Synergy**
***T-ALL #9791 (Unknown)***								
	Prednisolone	79 (45%)	Ruxolitinib	JAK1	ND (<20%)	NA	NA	NA
	Prednisolone	59 (38%)	AZD6244	MEK1/2	188 (48%)	NA	NA	**Synergy**
	Prednisolone	50 (40%)	Trametinib	MEK1/2	217 (76%)	**0.11 ± 0.08**	NA	**Strong synergy**
	Prednisolone	56 (41%)	MK2206	AKT	59 (59%)	0.81 ± 0.36	NA	No synergy
	Prednisolone	60 (43%)	AZD8055	mTOR	91 (90%)	**0.54 ± 0.18**	**0.41 ± 0.11**	**Synergy**
	Prednisolone	40 (44%)	Everolimus	mTOR	1 (50%)	NA	NA	NA
	Prednisolone	41 (46%)	NVPBEZ235	mTOR/PI3K	148 (89%)	**0.60 ± 0.31**	**0.61 ± 0.13**	**Synergy**

Rearrangements and mutations for patients are indicated. IC50 value is the inhibitory concentration of prednisolone or inhibitor at which 50% of total responding (in percent, between parentheses) leukemic cells died following 72 h of exposure. Based on IC50 values, synergy experiments were performed for prednisolone–inhibitor combinations at 1:1, 4:1, and 1:4 ratios. Mean CI values as a measure of cytotoxic synergy are indicated at effective dose levels ED50 and ED75 (including SD for triplicate experiments) for prednisolone–inhibitor combinations compared to single prednisolone or inhibitor response curves. For further details see also S9 Fig. CI = 1.00: additive effect; CI < 1.00: synergy; CI < 0.30: strong synergy; CI > 1.50: antagonism; synergy and strong synergy CI values given in bold.

*SD not given for CI values that were calculated on fewer than three prednisolone–inhibitor combinations.

CI, combination index; ETP-ALL, early thymic progenitor acute lymphoblastic leukemia; NA, not applicable; ND, not determined; ND (<20%), IC50 not determined when tested inhibitor had an efficacy < 20%; SD, standard deviation; T-ALL, T cell acute lymphoblastic leukemia.

## Discussion

Here, we report the identification of mutations in IL7R signaling molecules in 32% of pediatric patients with T-ALL. These mutations are associated with reduced steroid sensitivity and poor clinical outcome. In addition, we provide functional evidence that these mutations reduce steroid-induced cell death by activating the downstream signaling pathways MEK-ERK and AKT. Activation of these pathways causes (1) upregulation of the antiapoptotic proteins MCL1 and BCLXL; (2) inactivation of the proapoptotic protein BIM, an essential component in steroid-induced cell death; and (3) inactivation of GSK3B, a key regulator of BIM. Importantly, in cell lines and primary patient samples, inhibitors of IL7R signaling restored and enhanced steroid sensitivity, respectively.

WGS is a powerful technique used to identify breakpoint junctions of chromosomal rearrangements that result in the activation of oncogenes or create fusion proteins. It validated chromosomal translocations in all ten patients that were predicted by fluorescence in situ hybridization or other techniques, and led to the identification of various chromosomal rearrangements that had not been previously identified in T-ALL. Pediatric T-ALL patients on average have three (range 0–11) interchromosomal and 14 (range 5–25) intrachromosomal junctions as a consequence of translocations, inversions, deletions, duplications, or complex rearrangements. Multiple and clustered chromosomal junctions were identified in two patients that provided evidence for chromothripsis of Chromosomes 7 and 14 (ETP-ALL patient #10793) or Chromosomes 1 and 5 (proliferative T-ALL patient #10943). Most intra- and interchromosomal junctions, including those found in ETP-ALL patients, contain insertions of non-template-derived nucleotides, which strongly implies the involvement of the RAG1/2-mediated recombination machinery in these rearrangements.

Based on the WGS data for 13 patients and TES data from an expanded cohort of 69 T-ALL cases that were integrated with LOH data, we identified 151 mutated genes, of which two genes had not been observed before in human cancer (*RABL6* and *IGHV3-64*). Integrating this dataset with clinical and biological parameters identified IL7R signaling mutations as being associated with steroid resistance in pediatric T-ALL patients; these mutations may serve as biomarkers for reduced steroid response. Mutations affecting the IL7R signaling pathway and downstream AKT and MEK-ERK pathways were also associated with decreased relapse-free survival for pediatric T-ALL patients who had received DCOG or COALL treatment. Little is known about the prognostic significance of mutations in the IL7R-JAK/STAT pathway in T-ALL [49], and various studies have failed to demonstrate adverse effects for pediatric T-ALL patients harboring IL7R or IL7R/JAK mutations [50–52]. Our results are in line with the poor prognosis for patients with *JAK1* mutations originally reported for adult T-ALL [53] and with the poor prognosis for patients with *NRAS/KRAS* mutations reported for adult T-ALL patients treated in the GRAALL-2003 and GRAALL-2005 trials [54]. One explanation for varying results among studies may be that mutation rates for individual signaling components are low. To the best of our knowledge, no study has performed a comprehensive analysis of the prognostic significance of the full spectrum of IL7R signaling mutations in T-ALL before. These signaling mutations affect 32% of T-ALL patients, and are most recurrent in ETP-ALL patients ([40,52] and this study) and patients with the TLX subtype of T-ALL, which mostly harbors *HOXA*-activating aberrations or *TLX3* translocations [32].

Functional validation experiments in two T-ALL cell lines demonstrated that mutated IL7R signaling molecules robustly activate the downstream molecules AKT and MEK-ERK, thereby inducing steroid resistance. These include mutations in *IL7R* and *JAK1*, but not *JAK3*. Mutations in *JAK3* only modestly activate signaling, presumably due to their dependence on *JAK1* [55]. The IL7R signaling mutations do not affect NR3C1 signaling directly, including its nuclear translocation or its ability to transactivate target genes upon steroid exposure. These findings are consistent with our previous finding that steroid treatment activates NR3C1 target genes similarly between steroid-sensitive and steroid-resistant ALL patients [56], supporting the notion that resistance mechanisms are downstream—or independent—of steroid-induced NR3C1 transactivation. We found no evidence of differential NR3C1 serine 134 phosphorylation between the steroid-sensitive and steroid-resistant groups (NR3C1 serine 134 is phosphorylated by AKT and prevents nuclear translocation of NR3C1) [13]. We did not find increased MYB or BCL2 concentrations, previously proposed as steroid-resistance mechanisms in select xenograft ALL models [14].

Our data suggest that the NR3C1-driven proapoptotic response is reduced by an AKT/MEK-ERK-driven antiapoptotic response resulting in steroid resistance. AKT drives the expression of antiapoptotic MCL1 and BCLXL. In our resistant panel of cell lines, high MEK-ERK signaling was associated with the presence of phosphorylated (and inactivated) GSK3B and BIM, which are essential for steroid-induced death [43–46]. BIM is phosphorylated by ERK [57,58], and, in line with this, BIM was not phosphorylated in steroid-resistant lines upon treatment with the MEK inhibitor CI1040. GSK3B is an important regulator of proapoptotic BIM [48] and may prevent phosphorylation of BIM, thereby preserving steroid responsiveness. In addition to activated MEK-ERK, AKT can phosphorylate and inhibit GSK3B, as observed in the steroid-resistant panel of cell lines. Therefore, GSK3B may play an important role in the regulation of steroid responsiveness in general. Consistent with this, the GSK3B inhibitor IX strongly provoked steroid resistance in most steroid-sensitive cell lines. Our data therefore suggest that any cellular mechanism that may activate MEK-ERK and AKT can lead to steroid resistance in T-ALL, and may explain some steroid-resistant T-ALL cases that lack apparent IL7R signaling or NR3C1 mutations. The recently described NR3C1 cleavage by CASP1 might provide an alternative explanation for steroid resistance [19].

The finding that steroid resistance arises from activated MEK-ERK and AKT suggests that a therapeutic regime combining steroids with MEK, AKT, or mTOR inhibitors may increase steroid responsiveness. Importantly, these inhibitors might also increase the steroid response in steroid-sensitive patients. Interestingly, even though three of our patient samples harbored *IL7R* mutations, we found no proof of the effectiveness of ruxolitinib to reverse steroid resistance. This may possibly be due to a lack of cellular proliferation during in vitro drug sensitivity testing, and implies that dormant leukemic stem cells are not killed by ruxolitinib. Ruxolitinib has demonstrated some effectiveness in treating ETP-ALL in xenograft models [59,60], although a major effect was not achieved.

Our finding may also be highly relevant to BCP-ALL patients—who often have mutations in IL7R signaling molecules [61,62]—although this remains to be proven. High levels of AKT activity have been associated with steroid resistance and poor outcome in BCP-ALL [63]. Mutations in *NRAS* and *KRAS* are common in ALL and are associated with steroid resistance, central nervous system involvement, and poor outcome [16,17]; these mutations are preferentially acquired at relapse [18]. Connectivity mapping based on expression profiles of steroid-resistant ALL [64] suggests that mTOR inhibitors restore steroid sensitivity by reducing the levels of antiapoptotic MCL1 [65,66].

One possible limitation of our study is the relatively small cohort size. However, given the extremely low incidence of T-ALL (approximately 15 new patients identified in the Netherlands each year), it was not feasible to obtain larger numbers of clinically and biologically documented patient samples. A second limitation is that we were unable to obtain data regarding the effectiveness of inhibiting IL7R signaling inhibitors in vivo with respect to restoring steroid sensitivity in primary leukemic cells, e.g., using patient-derived T-ALL xenograft mouse models. In this respect, it is important to note that a recent preclinical study found that the PI3K/mTOR inhibitor NVPBEZ235 enhanced steroid sensitivity in a T-ALL xenograft model [67].

In conclusion, we provide evidence that steroid resistance in pediatric T-ALL patients is associated with mutations in IL7R signaling molecules. Because treatment success and clinical outcome are highly dependent upon steroid sensitivity in this setting, our findings suggest that small-molecule inhibitors of MEK, PI3K, AKT, and/or mTOR should be tested in order to restore or enhance steroid sensitivity in patients with ALL.

## Supporting Information

S1 Fig Filtering procedure for the WGS dataset from 13 pairs of diagnostic–remission patient samples. The filtering flowcharts to obtain candidate high-confidence somatic protein-altering SNVs (A) and INDELs (B) detected by WGS in 13 tumors are shown. INDEL, small insertion or deletion; SNV, single nucleotide variant; WGS, whole genome sequencing.
(TIF)

S2 Fig WGS quality threshold optimization by PCR–Sanger sequencing. (A) Scatterplot of somatic score (SS) versus total score (TS) for 46 mutations detected by WGS in 13 tumors and validated by PCR–Sanger sequencing. Mutations detected by PCR–Sanger sequencing only in the tumor and not in the remission sample are called true positives (TP, in green). Otherwise they are called false positives (FP, in red). The mutations that were not sequenced by PCR–Sanger sequencing are in grey. The entire space is divided into four quadrants using the thresholds SS = 0.1 and TS = 100, among which quadrant I is enriched for true positives. The different types of mutations are given by shape. del, small deletion; ins, small insertion; sub, small substitution; snv, single nucleotide variant. (B) Receiver operating characteristic (ROC) curves using SS and TS separately and jointly for 46 WGS mutations validated by PCR–Sanger sequencing. Three data points are marked where SS = 0.1 and TS = 100, separately and jointly.
(TIF)

S3 Fig TES quality threshold optimization exploiting the mutations detected by both TES and WGS in 13 tumor samples. (A) The training set and the classifier boundary for reliably detecting SNVs by TES. A mutation is called true positive (TP) if the exact mutation was found by both WGS and TES in the same patient sample. A mutation is called false positive (FP) if it was detected by TES, but not by WGS. The training set contains 570 exonic non-synonymous SNVs detected by TES in 13 tumor samples from the discovery cohort: 218 TP SNVs (green) versus 352 FP SNVs (red). The Gaussian one-class classifier boundary in blue results in false negative rate (FNr) = 0.10 and false positive rate (FPr) = 0.28. (B) The ROC curve of the Gaussian one-class classifier on the training set containing 570 TES SNVs from 13 tumor samples. The chosen boundary is indicated by the green circle on the curve, which has FNr = 0.10 and FPr = 0.28. (C) The training set and the classifier boundary for reliably detecting INDELs by TES. The training set contains 52 exonic non-synonymous INDELs detected by TES in 13 tumor samples from the discovery cohort: 11 TP INDELs (green) versus 41 FP INDELs (red). The classifier boundary in blue results in FNr = 0.10 and FPr = 0.63.
(TIF)

S4 Fig Filtering procedure for the TES dataset from 69 diagnostic patient samples. The filtering flowchart to obtain reliable somatic protein-altering SNVs (A) and INDELs (B) detected by targeted exome sequencing (TES) for 254 genes in 69 tumors is shown.
(TIF)

S5 Fig Activating IL7R signaling mutations confer steroid resistance. (A–C) Steroid response curves for steroid-sensitive SUPT1 cells that contain (A) IL7R, (B) non-cysteine mutant IL7R^GPSL^ or (C) cysteine mutant IL7R^RFCPH^ doxycycline-inducible lentiviral expression constructs. Steroid response curves are shown for induced (+Dox) or non-induced (−Dox) cells that have been exposed to serial dilutions (250–0.007 μg/ml) of prednisolone for 72 h. (D–F) Steroid response curves for steroid-sensitive P12 Ichikawa cells that contain (D) wild-type IL7R, (E) AKT^E17K^ or (F) wild-type AKT expression constructs. (G–I) Mean survival of P12 Ichikawa cells expressing wild-type or mutant IL7R signaling molecules (+Dox: open red bars) following a 72-h exposure to indicated concentrations of (G) prednisolone, (H) vincristine, or (I) *L*-asparaginase. Black bars represent the mean survival of all non-induced P12 Ichikawa lines following exposure to prednisolone, vincristine, or *L*-asparaginase (−Dox control). The steroid-sensitive panel refers to P12 Ichikawa lines that retain an equally sensitive steroid response compared to non-induced control conditions, whereas the steroid-resistant panel refers to lines that acquire steroid resistance. All data are from triplicate experiments and are represented as mean ± standard deviation.
(TIF)

S6 Fig IL7R signaling mutations do not affect the transactivation potential of NR3C1. GAPDH-normalized relative expression levels of (A) *NR3C1* (B) *TSC22D3/GILZ*, (C) *BCC3/PUMA*, (D) *KLF13*, (E) *BCL2L11/BIM*, and (F) *FKBP5* by real-time quantitative PCR in SUPT1 cell lines. Expression is displayed for all non-induced clones in the absence (−Dox, open black circles) or presence (−Dox, grey-filled black circles) of prednisolone, as well as for doxycycline-induced lines of the steroid-sensitive and -resistant panels in the absence (+Dox, open red circles) or presence (+Dox, grey-filled red circles) of prednisolone. Black bars indicate median expression levels.
(TIF)

S7 Fig Steroid resistance induced by wild-type or mutant IL7R signaling molecules is associated with activation of MEK-ERK and/or AKT. (A) Western blot results of total and/or phosphorylated levels of IL7R signaling molecules following induction (+Dox) of wild-type or mutant forms of IL7R, JAK1, JAK3, NRAS, and AKT molecules in SUPT1 cells. The steroid-sensitive and -resistant panels are indicated. Parental SUPT1 cells serve as a control. (B–N) Protein concentrations (as determined by β-actin-normalized band intensities) of (B) phospho-S^134^ NR3C1, (C) total RAS, (D) total PUMA, (E) total BAD, (F) phospho-BAD, (G) the phospho-GSK3B versus phospho-GSK3A ratio, (H) total BIM, (I) total BCL2, (J) phospho-CREB, (K) phospho-ATF1, (L) total MCL1, (M) phospho-p38, and (N) total cMYB in doxycycline-induced steroid-sensitive and -resistant SUPT1 lines. Significance levels were determined using the Mann-Whitney *U* test. Note that the total RAS levels in (C) are shown for all lines except RAS and NRAS^G12D^.
(TIF)

S8 Fig Reversal of steroid resistance by IL7R signaling inhibitors. (A) Activation status of IL7R signaling molecules by Western blot of SUPT1 cells expressing NRAS that were exposed to ruxolitinib (2 μM), CI1040 (10 μM), MK2206 (2 μM), or the CI1040/MK2206 combination for 24 h compared to non-induced and doxycycline-induced controls. (B–H) SUPT1 or P12 Ichikawa response curves to serial dilutions of prednisolone (250–0.007 μg/ml, 72 h exposure) without (−Dox, grey circles) or with (+Dox, open red circles) induction of (B) JAK1^R724H^, (C–E) NRAS, (F) IL7R, or (G and H) cysteine mutant IL7R^RFCPH^. The effect of (C, F, and G) 2 μM ruxolitinib, (D) 10 μM CI1040, or (E and H) 2 μM MK2206 on the steroid response under doxycycline-induced conditions is shown by open blue triangles. (I–K) Survival of P12 Ichikawa lines expressing wild-type or mutant IL7R signaling molecules following 72 h of treatment with 250 μg/ml prednisolone in the absence (open red bars) or presence (open blue bars) of the following inhibitors: (I) ruxolitinib, (J) CI1040, or (K) MK2206. The steroid-sensitive and -resistant panels are indicated. For each experiment, the mean survival percentage for all non-induced (+Dox) P12 Ichikawa lines following exposure to prednisolone (red, grey-filled bar) and prednisolone plus inhibitor (blue, grey-filled bar) is shown as a control. The survival of induced lines following exposure to steroids and inhibitors has been corrected for the cytotoxic effects of the inhibitors under non-induced control conditions. All data in (B–K) are from triplicate experiments and are represented as mean ± standard deviation.
(TIF)

S9 Fig Schematic overview of synergy experiments. (A) Single drug (i.e., prednisolone) or compound response curves for patient primary leukemic cells exposed to a 1–10,000 nM range of drug or inhibitor as indicated. IC50 values correspond to the drug or inhibitor concentrations at which 50% of responding cells have died within 72 h of exposure. (B) IC50 values as used for the synergy experiments for indicated prednisolone–inhibitor combinations. These experiments were performed for serial dilutions of prednisolone–inhibitor mixtures in a 1:1 ratio (the 10^0^ dilution equals [0.5 × IC50 prednisolone] + [0.5 × IC50 inhibitor]), a 1:4 ratio (the 10^0^ dilution equals [0.2 × IC50 prednisolone] + [0.8 × IC50 inhibitor]), and a 4:1 ratio (the 10^0^ dilution equals [0.8 × IC50 prednisolone] + [0.2 × IC50 inhibitor]). (C) Combination index (CI) as a measure for synergism or antagonism for each prednisolone–inhibitor combination mixture as calculated at the effective dose levels ED50 and ED75 (at which 50% and 75%, respectively, of the leukemic cells have died within 72 h of exposure) compared to the corresponding single prednisolone and inhibitor response curves (for which the 10^0^ dilution equals the IC50 concentration of prednisolone or inhibitor). CI = 1.0 indicates additive effects; CI < 1.0, synergistic effects; CI < 0.3, strong synergistic effects; and CI > 1.5, antagonistic effects. When CI = 0.1, the prednisolone–inhibitor combination achieves a similar cytotoxic effect at 10-fold lower concentrations than estimated from the prednisolone or inhibitor single response curves.
(TIF)

S1 Table Summary of T-ALL patients included in this study. Treatment protocols and available genomic and transcriptomic data are indicated.
(XLSX)

S2 Table Discovery cohort of diagnostic–remission samples of 13 pediatric T-ALL patients for whole genome sequencing. (XLSX)

S3 Table Expansion cohort for targeted exome sequencing: additional 69 COALL-97/03 pediatric T-ALL cohort diagnostic samples. (XLSX)

S4 Table Biological pathway enrichment analysis of 127 recurrently mutated genes in the expansion cohort. (XLSX)

S5 Table Association between mutations/aberrations and poor outcome, drug resistance, and T-ALL subtype with nominal *p <* 0.05 in the cohort of 69 T-ALL patients in the expansion phase. (XLSX)

S6 Table Overview of mutations and aberrations in IL7R signaling pathway genes in 146 pediatric T-ALL patients. (XLSX)

## Abbreviations

aCGH: array comparative genomic hybridization
ALL: acute lymphoblastic leukemia
BCP-ALL: B cell precursor acute lymphoblastic leukemia
CI: combination index
COALL: German Co-operative Study Group for Childhood Acute Lymphoblastic Leukemia
DCOG: Dutch Childhood Oncology Group
ETP-ALL: early thymic progenitor acute lymphoblastic leukemia
FNr: false negative rate
FPr: false positive rate
INDEL: small insertion or deletion
LOH: loss of heterozygosity
ROC: receiver operating characteristic
SNV: single nucleotide variant
SS: somatic score
T-ALL: T cell acute lymphoblastic leukemia
TES: targeted exome sequencing
TS: total score
WGS: whole genome sequencing
