# Supplementary figures and images for: IL-7 Receptor Mutations and Steroid Resistance in Pediatric T cell Acute Lymphoblastic Leukemia: A Genome Sequencing Study
Source: PLoS Med. 2016 Dec 20;13(12):e1002200. doi: 10.1371/journal.pmed.1002200 (PMC5172551; doi:10.1371/journal.pmed.1002200)

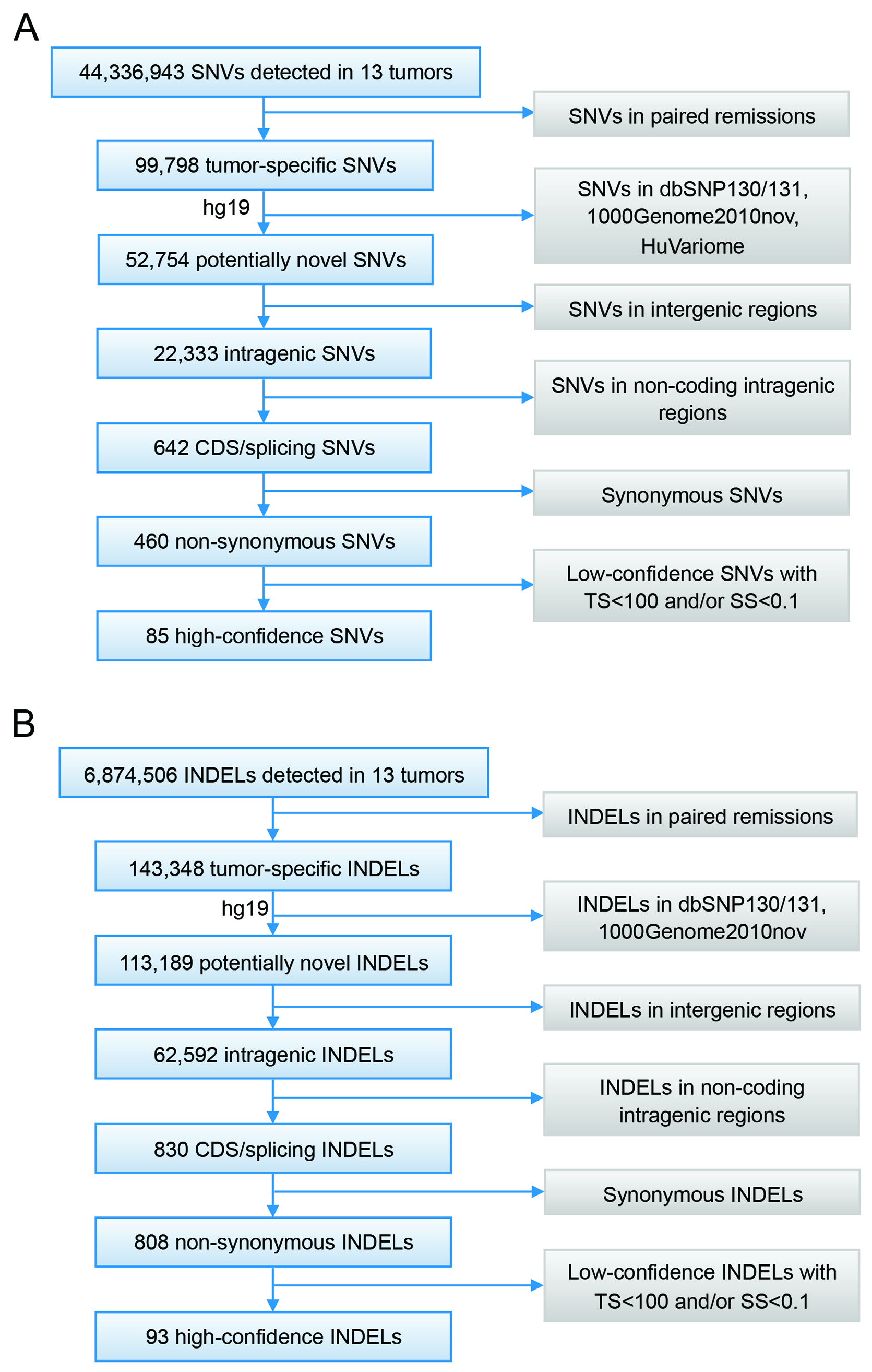

Supplement: S1 Fig — The filtering flowcharts to obtain candidate high-confidence somatic protein-altering SNVs (A) and INDELs (B) detected by WGS in 13 tumors are shown. INDEL, small insertion or deletion; SNV, single nucleotide variant; WGS, whole genome sequencing. (TIF) [file pmed.1002200.s001.tif]

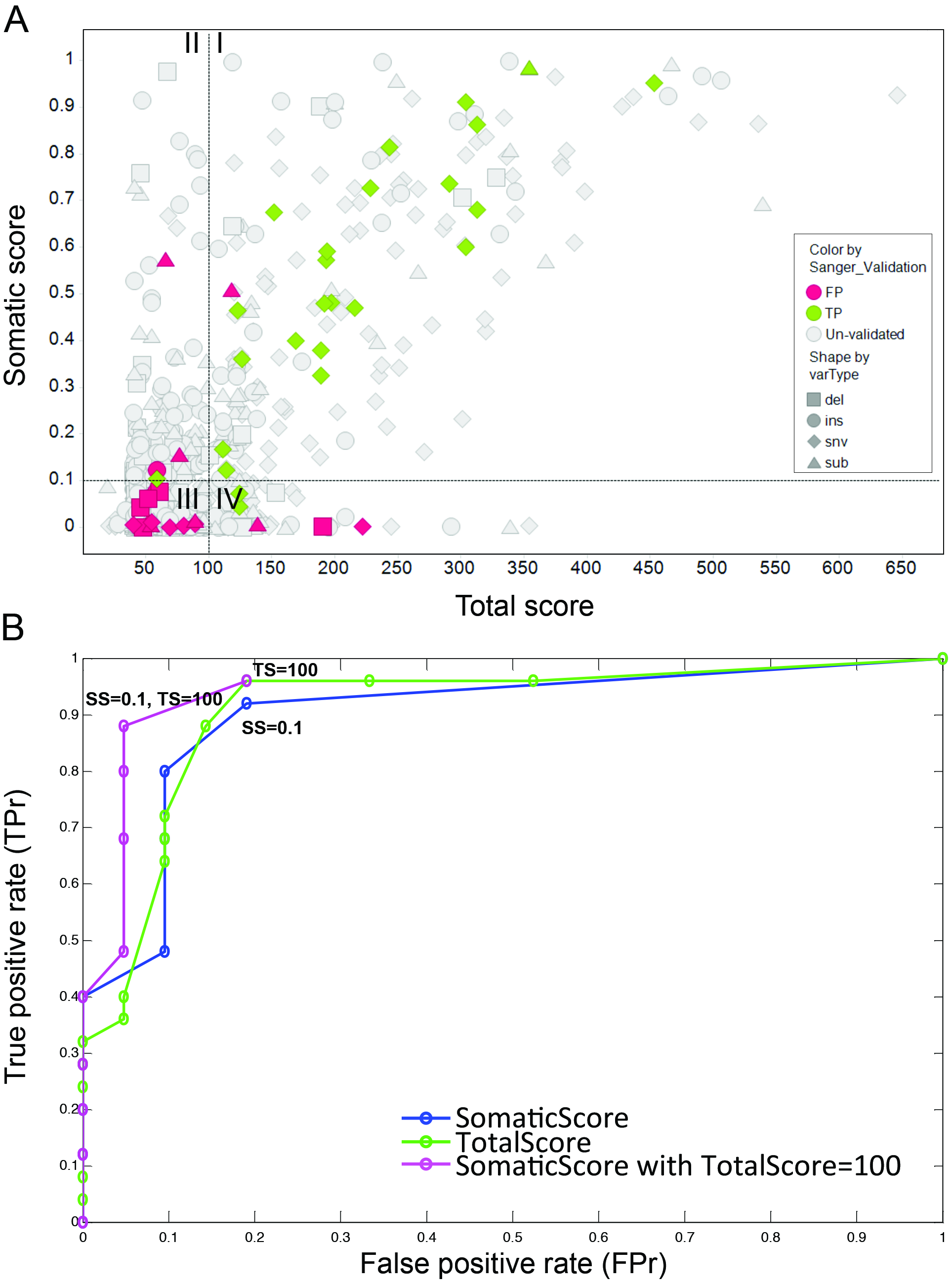

Supplement: S2 Fig — (A) Scatterplot of somatic score (SS) versus total score (TS) for 46 mutations detected by WGS in 13 tumors and validated by PCR–Sanger sequencing. Mutations detected by PCR–Sanger sequencing only in the tumor and not in the remission sample are called true positives (TP, in green). Otherwise they are called false positives (FP, in red). The mutations that were not sequenced by PCR–Sanger sequencing are in grey. The entire space is divided into four quadrants using the thresholds SS = 0.1 and TS = 100, among which quadrant I is enriched for true positives. The different types of mutations are given by shape. del, small deletion; ins, small insertion; sub, small substitution; snv, single nucleotide variant. (B) Receiver operating characteristic (ROC) curves using SS and TS separately and jointly for 46 WGS mutations validated by PCR–Sanger sequencing. Three data points are marked where SS = 0.1 and TS = 100, separately and jointly. (TIF) [file pmed.1002200.s002.tif]

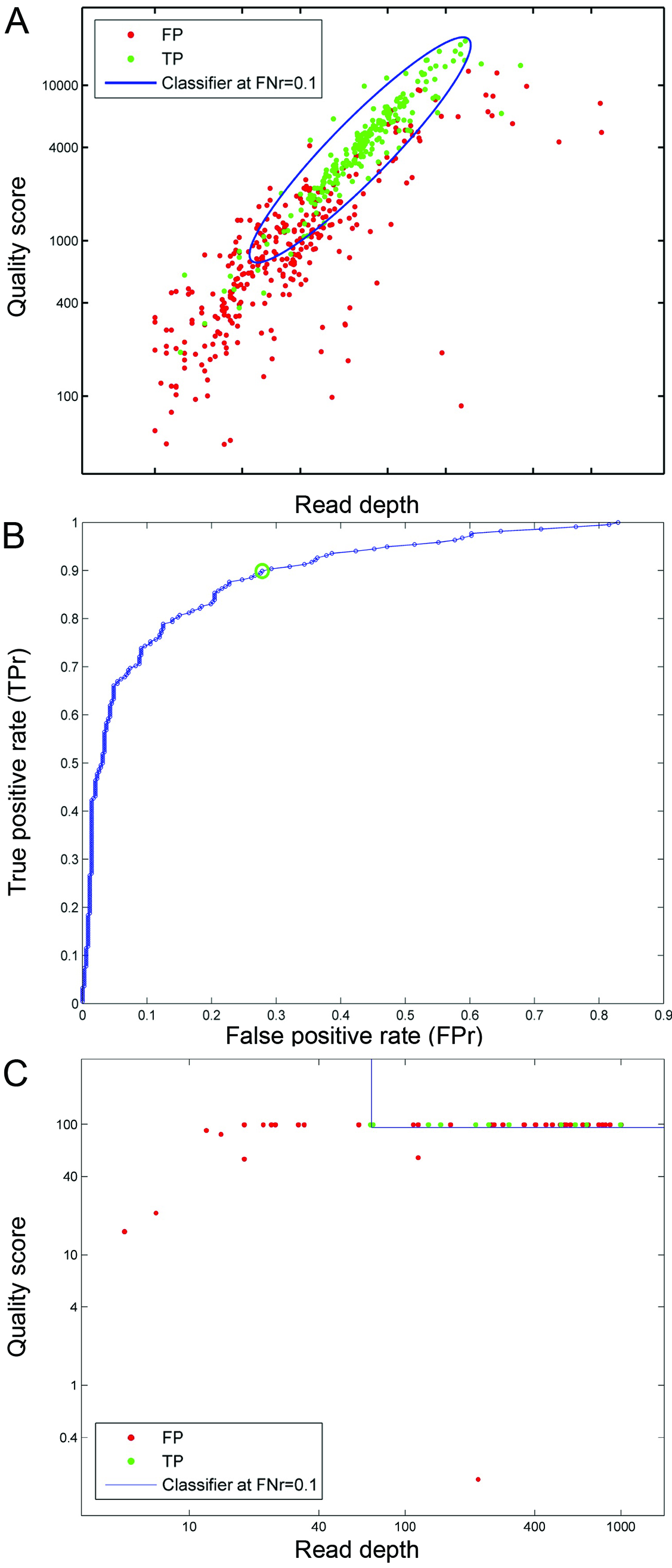

Supplement: S3 Fig — (A) The training set and the classifier boundary for reliably detecting SNVs by TES. A mutation is called true positive (TP) if the exact mutation was found by both WGS and TES in the same patient sample. A mutation is called false positive (FP) if it was detected by TES, but not by WGS. The training set contains 570 exonic non-synonymous SNVs detected by TES in 13 tumor samples from the discovery cohort: 218 TP SNVs (green) versus 352 FP SNVs (red). The Gaussian one-class classifier boundary in blue results in false negative rate (FNr) = 0.10 and false positive rate (FPr) = 0.28. (B) The ROC curve of the Gaussian one-class classifier on the training set containing 570 TES SNVs from 13 tumor samples. The chosen boundary is indicated by the green circle on the curve, which has FNr = 0.10 and FPr = 0.28. (C) The training set and the classifier boundary for reliably detecting INDELs by TES. The training set contains 52 exonic non-synonymous INDELs detected by TES in 13 tumor samples from the discovery cohort: 11 TP INDELs (green) versus 41 FP INDELs (red). The classifier boundary in blue results in FNr = 0.10 and FPr = 0.63. (TIF) [file pmed.1002200.s003.tif]

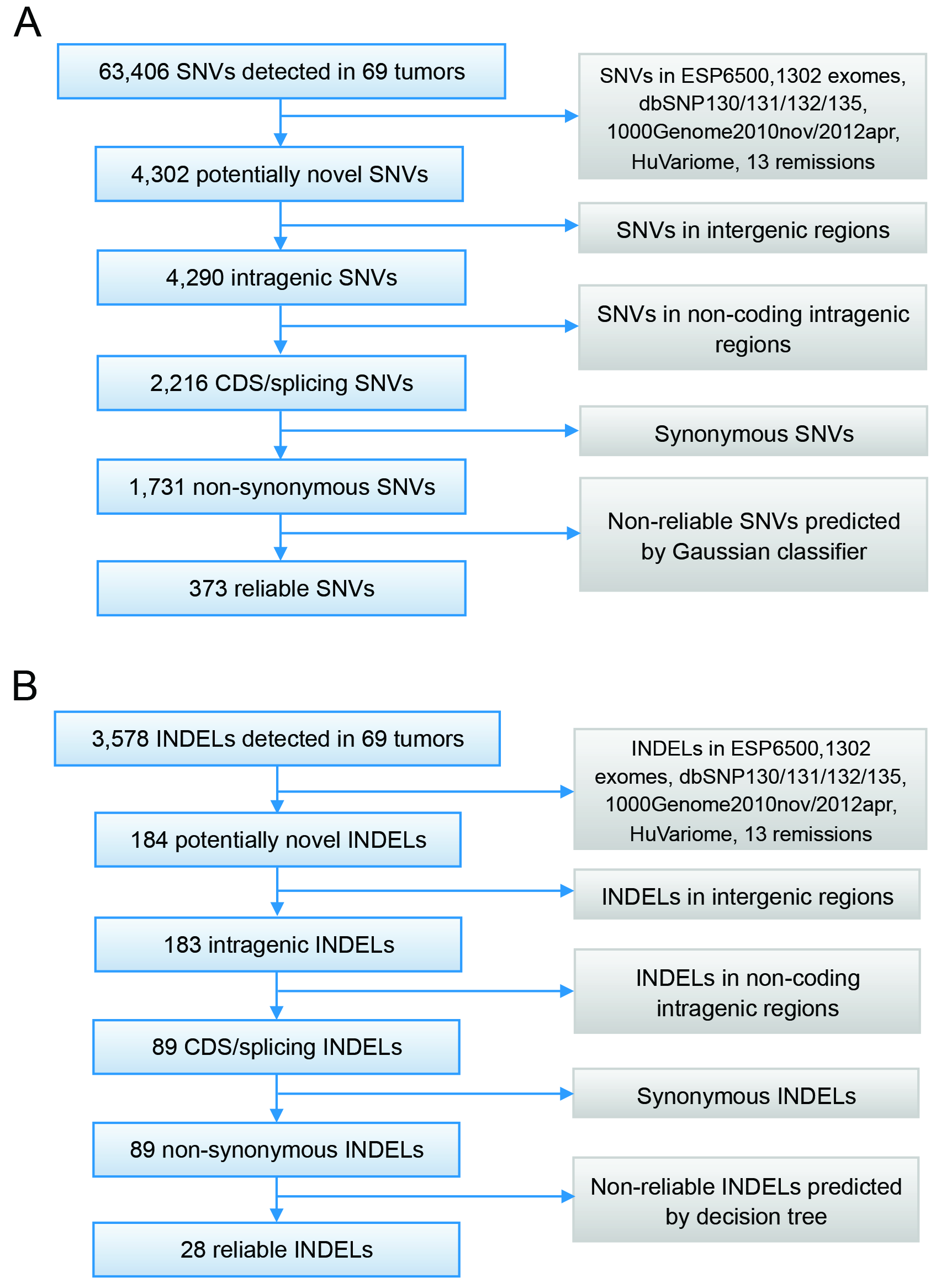

Supplement: S4 Fig — The filtering flowchart to obtain reliable somatic protein-altering SNVs (A) and INDELs (B) detected by targeted exome sequencing (TES) for 254 genes in 69 tumors is shown. (TIF) [file pmed.1002200.s004.tif]

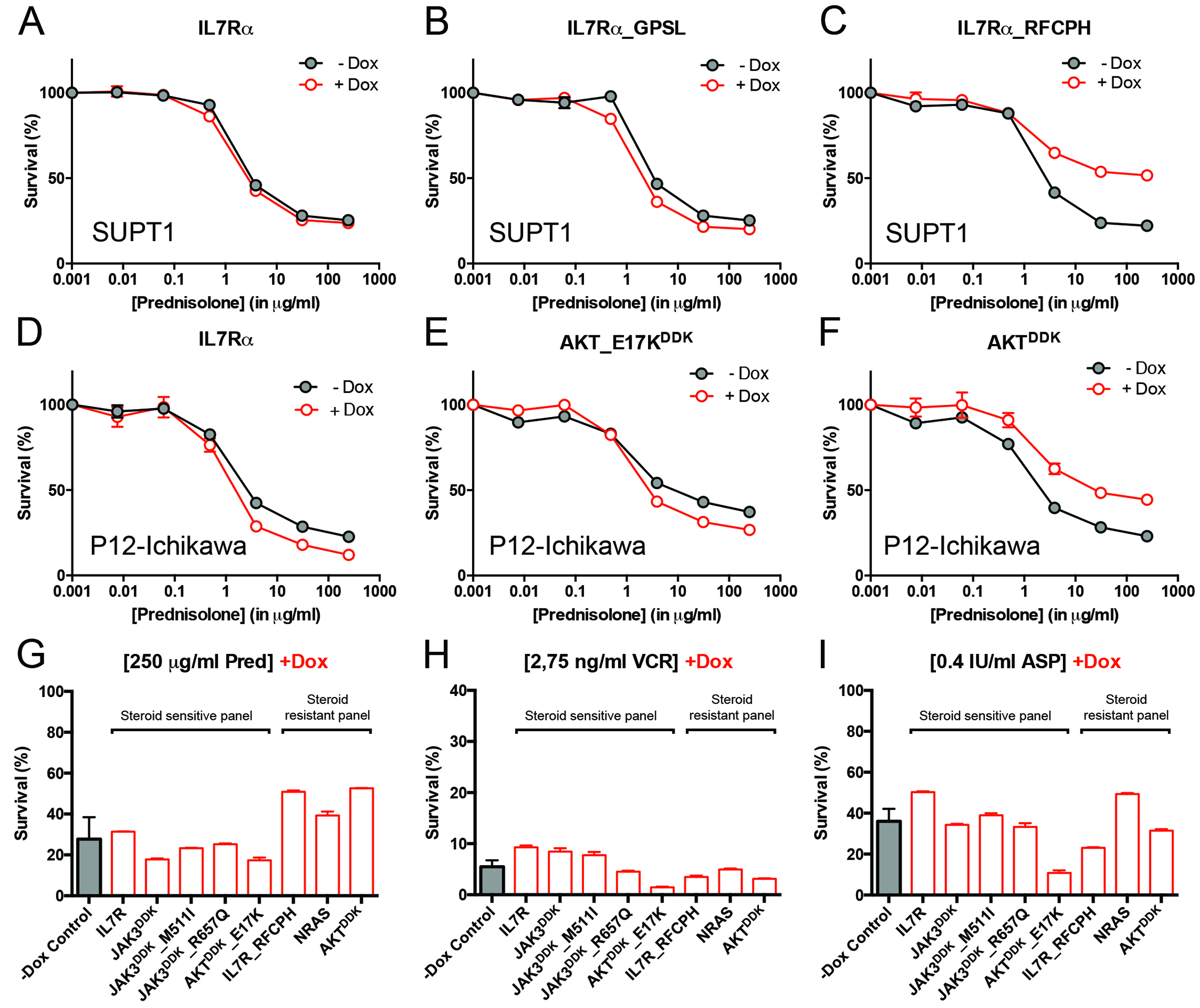

Supplement: S5 Fig — (A–C) Steroid response curves for steroid-sensitive SUPT1 cells that contain (A) IL7R, (B) non-cysteine mutant IL7RGPSL or (C) cysteine mutant IL7RRFCPH doxycycline-inducible lentiviral expression constructs. Steroid response curves are shown for induced (+Dox) or non-induced (−Dox) cells that have been exposed to serial dilutions (250–0.007 μg/ml) of prednisolone for 72 h. (D–F) Steroid response curves for steroid-sensitive P12 Ichikawa cells that contain (D) wild-type IL7R, (E) AKTE17K or (F) wild-type AKT expression constructs. (G–I) Mean survival of P12 Ichikawa cells expressing wild-type or mutant IL7R signaling molecules (+Dox: open red bars) following a 72-h exposure to indicated concentrations of (G) prednisolone, (H) vincristine, or (I) L-asparaginase. Black bars represent the mean survival of all non-induced P12 Ichikawa lines following exposure to prednisolone, vincristine, or L-asparaginase (−Dox control). The steroid-sensitive panel refers to P12 Ichikawa lines that retain an equally sensitive steroid response compared to non-induced control conditions, whereas the steroid-resistant panel refers to lines that acquire steroid resistance. All data are from triplicate experiments and are represented as mean ± standard deviation. (TIF) [file pmed.1002200.s005.tif]

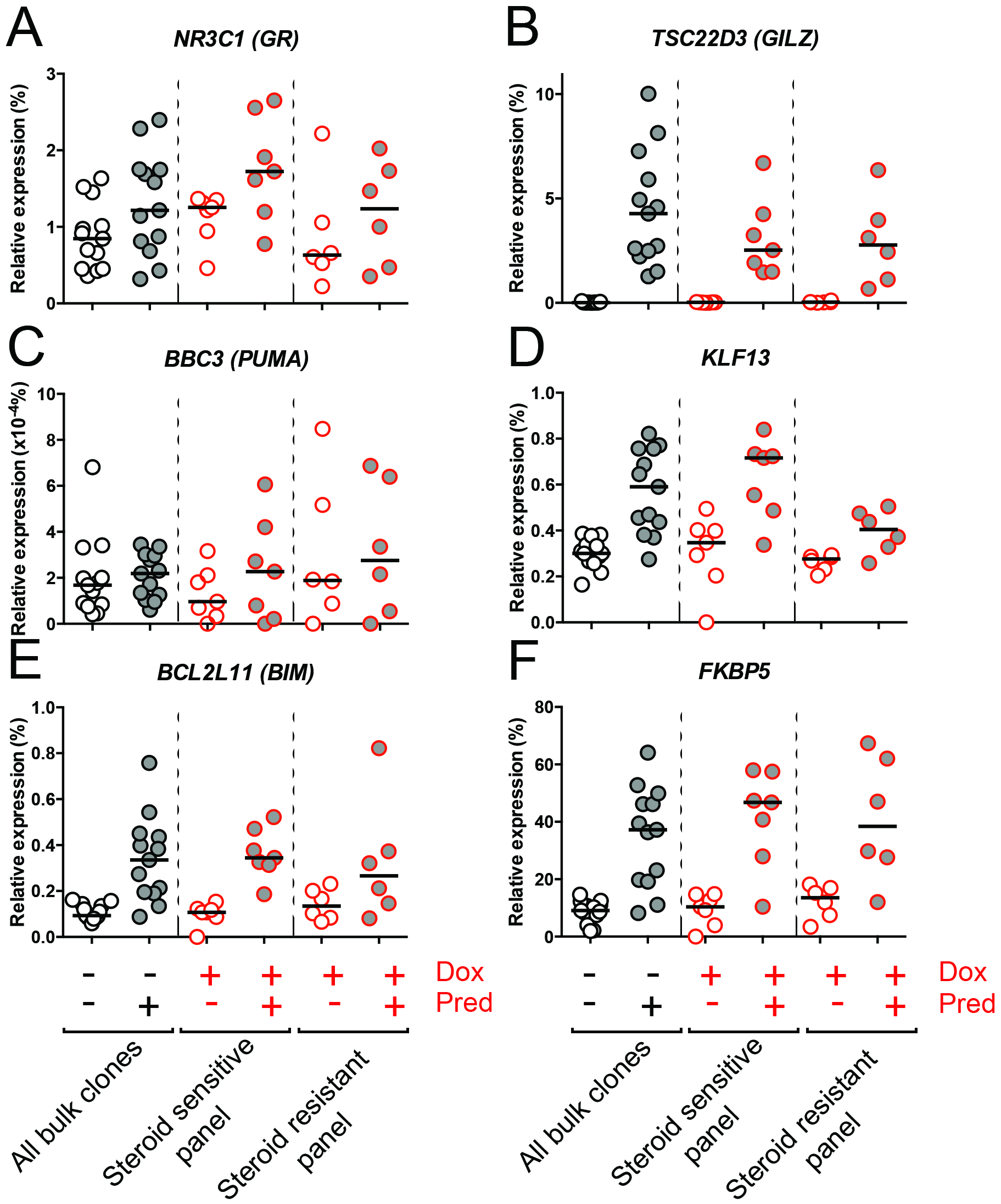

Supplement: S6 Fig — GAPDH-normalized relative expression levels of (A) NR3C1 (B) TSC22D3/GILZ, (C) BCC3/PUMA, (D) KLF13, (E) BCL2L11/BIM, and (F) FKBP5 by real-time quantitative PCR in SUPT1 cell lines. Expression is displayed for all non-induced clones in the absence (−Dox, open black circles) or presence (−Dox, grey-filled black circles) of prednisolone, as well as for doxycycline-induced lines of the steroid-sensitive and -resistant panels in the absence (+Dox, open red circles) or presence (+Dox, grey-filled red circles) of prednisolone. Black bars indicate median expression levels. (TIF) [file pmed.1002200.s006.tif]

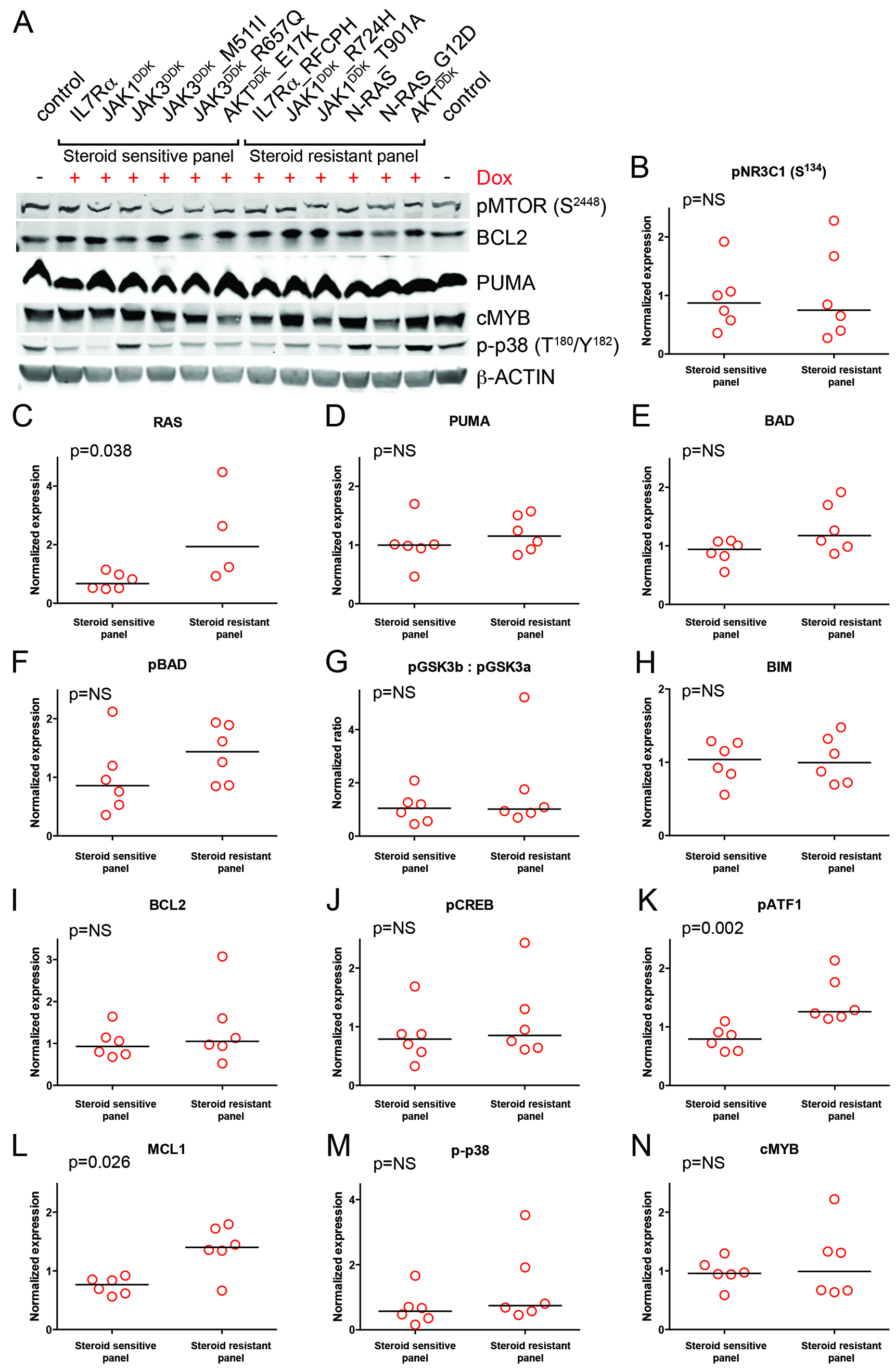

Supplement: S7 Fig — (A) Western blot results of total and/or phosphorylated levels of IL7R signaling molecules following induction (+Dox) of wild-type or mutant forms of IL7R, JAK1, JAK3, NRAS, and AKT molecules in SUPT1 cells. The steroid-sensitive and -resistant panels are indicated. Parental SUPT1 cells serve as a control. (B–N) Protein concentrations (as determined by β-actin-normalized band intensities) of (B) phospho-S134 NR3C1, (C) total RAS, (D) total PUMA, (E) total BAD, (F) phospho-BAD, (G) the phospho-GSK3B versus phospho-GSK3A ratio, (H) total BIM, (I) total BCL2, (J) phospho-CREB, (K) phospho-ATF1, (L) total MCL1, (M) phospho-p38, and (N) total cMYB in doxycycline-induced steroid-sensitive and -resistant SUPT1 lines. Significance levels were determined using the Mann-Whitney U test. Note that the total RAS levels in (C) are shown for all lines except RAS and NRASG12D. (TIF) [file pmed.1002200.s007.tif]

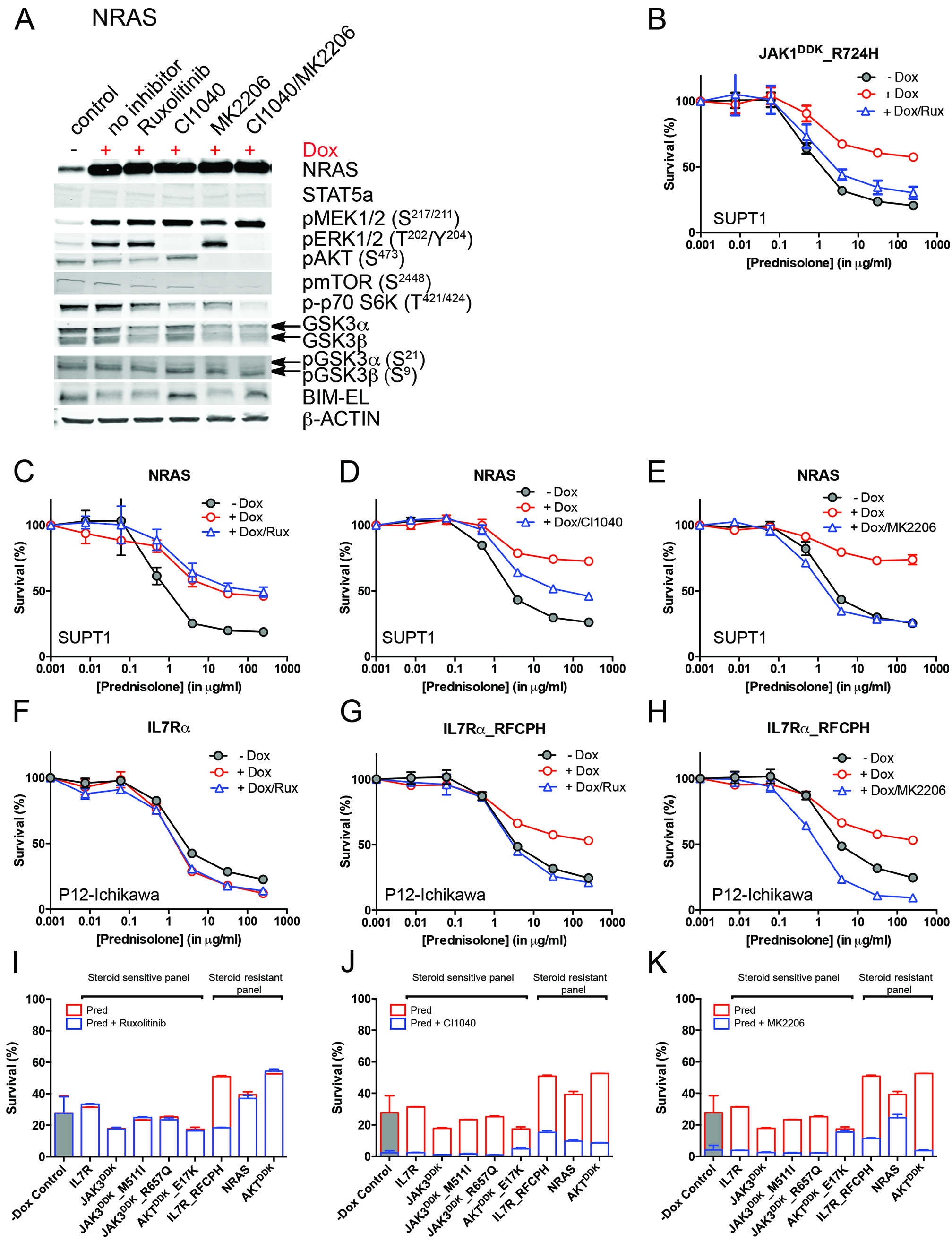

Supplement: S8 Fig — (A) Activation status of IL7R signaling molecules by Western blot of SUPT1 cells expressing NRAS that were exposed to ruxolitinib (2 μM), CI1040 (10 μM), MK2206 (2 μM), or the CI1040/MK2206 combination for 24 h compared to non-induced and doxycycline-induced controls. (B–H) SUPT1 or P12 Ichikawa response curves to serial dilutions of prednisolone (250–0.007 μg/ml, 72 h exposure) without (−Dox, grey circles) or with (+Dox, open red circles) induction of (B) JAK1R724H, (C–E) NRAS, (F) IL7R, or (G and H) cysteine mutant IL7RRFCPH. The effect of (C, F, and G) 2 μM ruxolitinib, (D) 10 μM CI1040, or (E and H) 2 μM MK2206 on the steroid response under doxycycline-induced conditions is shown by open blue triangles. (I–K) Survival of P12 Ichikawa lines expressing wild-type or mutant IL7R signaling molecules following 72 h of treatment with 250 μg/ml prednisolone in the absence (open red bars) or presence (open blue bars) of the following inhibitors: (I) ruxolitinib, (J) CI1040, or (K) MK2206. The steroid-sensitive and -resistant panels are indicated. For each experiment, the mean survival percentage for all non-induced (+Dox) P12 Ichikawa lines following exposure to prednisolone (red, grey-filled bar) and prednisolone plus inhibitor (blue, grey-filled bar) is shown as a control. The survival of induced lines following exposure to steroids and inhibitors has been corrected for the cytotoxic effects of the inhibitors under non-induced control conditions. All data in (B–K) are from triplicate experiments and are represented as mean ± standard deviation. (TIF) [file pmed.1002200.s008.tif]

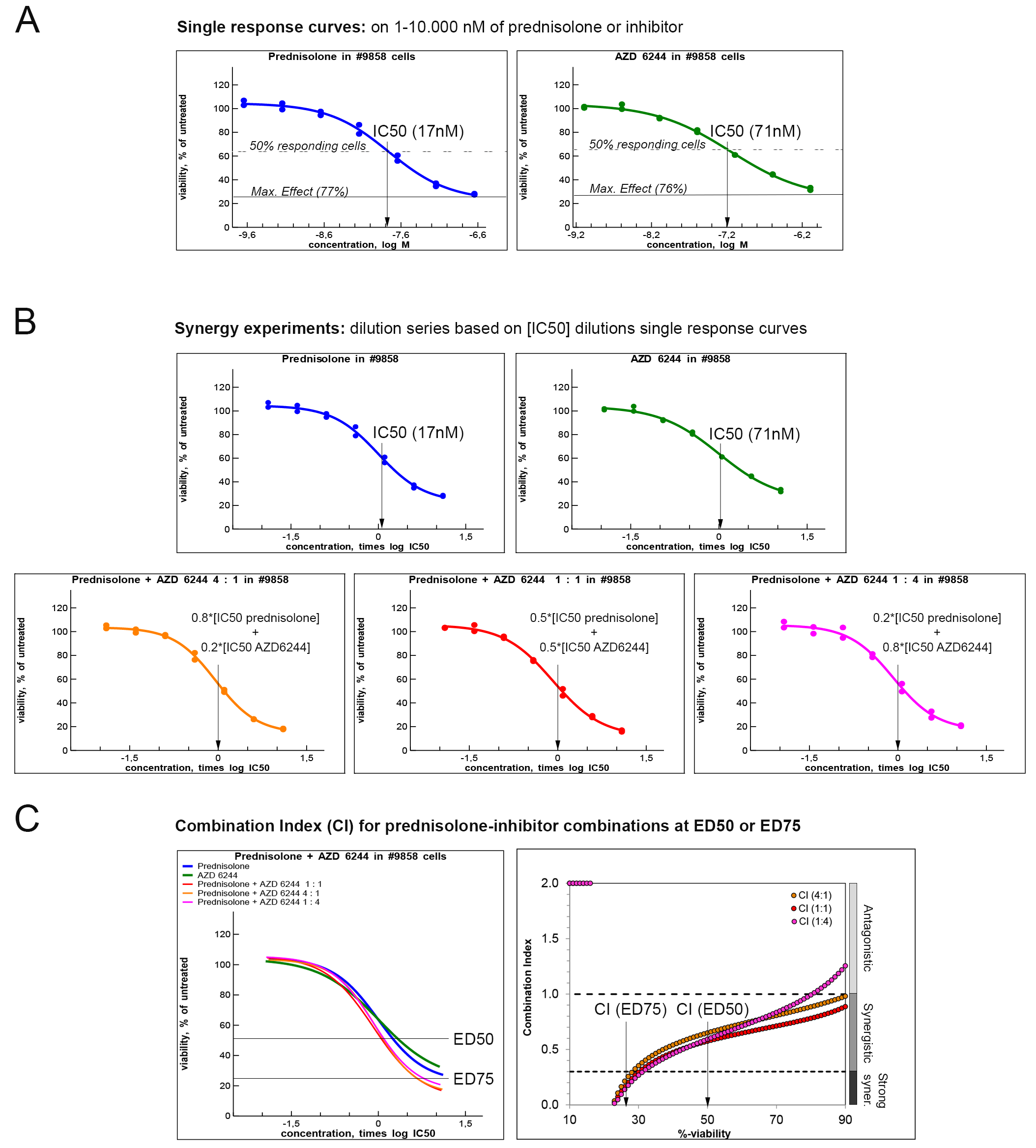

Supplement: S9 Fig — (A) Single drug (i.e., prednisolone) or compound response curves for patient primary leukemic cells exposed to a 1–10,000 nM range of drug or inhibitor as indicated. IC50 values correspond to the drug or inhibitor concentrations at which 50% of responding cells have died within 72 h of exposure. (B) IC50 values as used for the synergy experiments for indicated prednisolone–inhibitor combinations. These experiments were performed for serial dilutions of prednisolone–inhibitor mixtures in a 1:1 ratio (the 100 dilution equals [0.5 × IC50 prednisolone] + [0.5 × IC50 inhibitor]), a 1:4 ratio (the 100 dilution equals [0.2 × IC50 prednisolone] + [0.8 × IC50 inhibitor]), and a 4:1 ratio (the 100 dilution equals [0.8 × IC50 prednisolone] + [0.2 × IC50 inhibitor]). (C) Combination index (CI) as a measure for synergism or antagonism for each prednisolone–inhibitor combination mixture as calculated at the effective dose levels ED50 and ED75 (at which 50% and 75%, respectively, of the leukemic cells have died within 72 h of exposure) compared to the corresponding single prednisolone and inhibitor response curves (for which the 100 dilution equals the IC50 concentration of prednisolone or inhibitor). CI = 1.0 indicates additive effects; CI < 1.0, synergistic effects; CI < 0.3, strong synergistic effects; and CI > 1.5, antagonistic effects. When CI = 0.1, the prednisolone–inhibitor combination achieves a similar cytotoxic effect at 10-fold lower concentrations than estimated from the prednisolone or inhibitor single response curves. (TIF) [file pmed.1002200.s009.tif]
